# Targeting KDM4 family epigenetically triggers antitumour immunity via enhancing tumour‐intrinsic innate sensing and immunogenicity

**DOI:** 10.1002/ctm2.1598

**Published:** 2024-02-23

**Authors:** Mayu Sun, Xiaoyu Han, Jinyang Li, Jiali Zheng, Jingquan Li, Hui Wang, Xiaoguang Li

**Affiliations:** ^1^ State Key Laboratory of Systems Medicine for Cancer Center for Single‐Cell Omics School of Public Health Shanghai Jiao Tong University School of Medicine Shanghai China; ^2^ Department of Epidemiology and Biostatistics, School of Public Health Shanghai Jiao Tong University School of Medicine Shanghai China

**Keywords:** immunogenicity, JIB‐04, KDM4, tumour‐intrinsic innate sensing, type I interferons

## Abstract

Despite the remarkable clinical efficacy of cancer immunotherapy, considerable patients fail to benefit from it due to primary or acquired resistance. Tumours frequently hijack diverse epigenetic mechanisms to evade immune detection, thereby highlighting the potential for pharmacologically targeting epigenetic regulators to restore the impaired immunosurveillance and re‐sensitise tumours to immunotherapy. Herein, we demonstrated that KDM4‐targeting chemotherapeutic drug JIB‐04, epigenetically triggered the tumour‐intrinsic innate immune responses and immunogenic cell death (ICD), resulting in impressive antitumour effects. Specifically, JIB‐04 induced H3K9 hypermethylation through specific inhibition of the KDM4 family (KDM4A–D), leading to impaired DNA repair signalling and subsequent DNA damage. As a result, JIB‐04 not only activated the tumour‐intrinsic cyclic GMP‐AMP synthase (cGAS)‐STING pathway via DNA‐damage‐induced cytosolic DNA accumulation, but also promoted ICD, releasing numerous damage‐associated molecular patterns. Furthermore, JIB‐04 induced adaptive resistance through the upregulation of programmed death‐ligand 1 (PD‐L1), which could be overcome with additional PD‐L1 blockade. In human tumours, KDM4B expression was negatively correlated with clinical outcomes, type I interferon signatures, and responses to immunotherapy. In conclusion, our results demonstrate that targeting KDM4 family can activate tumour‐intrinsic innate sensing and immunogenicity, and synergise with immunotherapy to improve antitumour outcomes.

## Graphical abstract

1. JIB‐04 activates type I IFN signaling in both human and mouse cancer cells.
2. JIB‐04 enhances tumor‐intrinsic DNA sensing and immunogenicity through specific inhibition of the KDM4 family.
3. Targeting KDM4 family triggers innate and adaptive antitumor immunity.

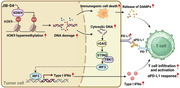

## 1 BACKGROUND

Immune evasion constitutes a principal hallmark of cancer, and the re‐establishment of immune surveillance has been proven to be a potent antitumour strategy.^1^ Immune checkpoint inhibitors (ICIs) can reprogram the immune system to launch powerful and targeted attacks on cancer cells, leading to impressive clinical outcomes in various cancer types.^2^ However, less than 30% of patients derive objective responses to ICIs.^3^, ^4^ Lack of efficient antitumour T‐cell immunity, also known as “immune‐cold” tumours, characterises majority of non‐responsive patients.^5^ Combining various immune‐based therapies to turn cold tumours into T‐cell‐inflamed tumours exhibits tremendous potential to expand the benefits of immunotherapies to less favourable immune phenotypes.^6^

Tumour microenvironment (TME) that is inflamed by T cells is mainly identified by a signature of type I interferon (IFN), which is an essential element of innate immunity.^7^ Multiple cell populations, including tumour cells and dendritic cells, produce type I IFNs upon activation by viral constituents, pathogen‐associated molecular patterns or DNA fragments emitted by dying cells.^8^ Type I IFNs are instrumental in facilitating dendritic cell cross‐presentation, as well as directly bolstering the functions of natural killer and T cells, thereby connecting innate and adaptive immunity within the TME.^9^ Extensive studies have demonstrated a strong correlation between heightened type I IFN signalling within tumour cells and improved T‐cell abundance and efficacy in the TME.^9^, ^10^ Furthermore, the full effectiveness of a broad range of anticancer treatments, such as chemotherapy, targeted therapies, and immunotherapies, depends significantly on the essential role of type I IFN signalling.^8^ Nevertheless, the mechanisms responsible for the functional suppression of type I IFN synthesis in the majority of tumours remain largely unidentified.^11^, ^12^, ^13^

Epigenetic mechanisms are important not only in the initiation and advancement of cancer, but also in tumour immunity. Recent studies in cancer epigenetics have revealed several epigenomic modulators involved in chromatin remodeling, histone modification, DNA methylation and RNA modification, which have a significant impact on tumour immunogenicity, functions of immune cells and immunotherapy response.^14^, ^15^, ^16^, ^17^ Therefore, therapeutic strategies involving epigenetic‐modifying drugs hold promise for synergistically enhance tumour immunogenicity by stimulating the production of transcriptionally silenced tumour antigens, promoting the presence and presentation of neoantigens, and activating tumour immunogenic cell death (ICD).^15^

In this study, we aimed to discovery potent epigenetic modulators that could serve as effective elements in immune‐based combination therapy, leading to improved therapeutic outcomes. We provided evidence that histone lysine demethylases (KDM) are pivotal in suppressing type I IFN response within tumour cells. Specifically, we identified JIB‐04, a pan‐inhibitor of KDM4, as a powerful inducer of antitumour T‐cell immunity. By targeting KDM4 family, JIB‐04 triggered tumour‐intrinsic innate sensing to secrete type I IFNs and promoted ICD, ultimately improving antitumour innate and adaptive immunity. Furthermore, anti‐programmed death‐ligand 1 (PD‐L1) immunotherapy could effectively counteract the adaptive immune resistance induced by KDM4 inhibition, resulting in remarkable antitumour effects. Our findings provide valuable information about the function of KDM4 in controlling the innate sensing and immunogenicity of tumours, and propose a strong basis for combining KDM4 inhibition with anti‐PD‐1/PD‐L1 immunotherapy in cancer patients.

## 2 METHODS AND MATERIALS

### 2.1 Mice and tumour cell lines

Female C57BL/6 mice (5–6 weeks) were obtained from Shanghai Lingchang Biotechnology and housed under pathogen‐free conditions. All animal protocols were conducted in approvement with the Institutional Animal Care and Use Committee at the Shanghai Jiao Tong University School of Medicine. Murine tumour cells MC38, B16, CT26, Panc02 and 4T1, and human cells SW1116, SW480, SW620, A375, MDA‐MB‐231 and MDA231‐LM2‐4175 were purchased from American Type Culture Collection or National Collection of Authenticated Cell cultures of the Shanghai Institutes for Biological Sciences. The CTIBA5 cell line was generously provided by Dr. Rolf Brekken from the University of Texas Southwestern Medical Center. All cell lines were confirmed to be free of mycoplasma contamination.

### 2.2 Reagents

The reagents included JIB‐04 (TargetMol, cat#199596‐05‐09), cisplatin (MedChemExpress, cat#15663‐27‐1), poly(I:C) (Invivogen, cat#tlrl‐picwlv) and TACH101 (ProbeChem, cat#PC‐7225). Anti‐CD8 antibody (cat#BE0118), anti‐IFNAR‐1 antibody (cat#BP0241), mouse IgG2a isotype control (cat#BE0085), rat IgG2b isotype control (cat#BE0090) and anti‐PD‐L1 antibody (cat#BE0101) were purchased from BioXcell and applied for in vivo assay.

#### 2.2.1 In vitro high‐throughput drug screening

To discover novel drugs capable of inducing type I IFN secretion, we performed a high‐throughput screen using a commercial anti‐cancer compound library obtained from TargeMol (cat#L1200). First, 30 000 cells Raw‐Lucia ISG cells (InvivoGen) were plated in 96‐well plates. Once attached, the cells were exposed with 1 μM of the compounds diluted in RPMI and incubated at 37°C for a period of 24 h. Post‐incubation, we transferred the culture supernatant to a new 96‐well plate and added QUANTI‐Luc 4 Lucia/gauss for luciferase activity measurement, which was then immediately read using a microplate reader.

### 2.3 Generation of CRISPR‐edited cells

The sgRNA sequences were designed using the CRISPR designing tool, and are listed in Table S1. The knockout cells were generated as previously described.^18^

### 2.4 Mouse tumour models

MC38 (1 × 10^6^ cells/mouse) or B16 (5 × 10^5^ cells/mouse) cells were subcutaneously implanted into the flank of female mice. Tumour volume calculations were based on the formula of length × width^2^/2, with euthanasia scheduled upon reaching a tumour size of 2000 mm^3^. JIB‐04 was dissolved in Dimethyl sulfoxide (DMSO) and diluted further with phosphate‐buffered saline (PBS) containing 10% Kolliphor EL. Mice were grouped randomly and injected intratumourally with JIB‐04 (.57 mg per injection) every day for a total of four to seven times. The control group received an equal volume solvent. To block PD‐1/PD‐L1 signalling, anti‐PD‐L1 antibodies (50 μg per mouse for MC38 tumours, 100 μg per mouse for B16 tumours) were intraperitoneally administered every 4 days. Blockade of the IFN‐α/β receptor involved intratumoural administration of 150 μg of antibodies against IFNAR1 every 3 days. Depletion of CD8^+^ T cells was achieved through intraperitoneal administration of 100 μg of anti‐mouse CD8 antibodies every 3 days.

### 2.5 Quantitative real‐time polymerase chain reaction

To extract total RNA, the Direct‐zol RNA Miniprep Plus Kit (R2070, ZTMO RESARCH) was used to lyse tumour cells or tissues. Subsequently, the isolated RNA was reverse transcribed into cDNA. Quantitative real‐time polymerase chain reaction (RT‐qPCR) was performed and gene expression was quantified using the 2^−ΔΔCT^ method normalised against actin. Table S2 provides a list of the RT‐qPCR primers.

### 2.6 Enzyme‐linked immunosorbent assays

To measure IFN‐β levels, the supernatant from JIB‐04‐treated tumour cells underwent a two‐step centrifugation process. At first, the sample was subjected to centrifugation to obtain cell‐free supernatant. The mouse IFN‐β ELISA kit (PBL Assay Science, cat#42410) was used to quantify the concentration of IFN‐β, following the instruction manual. In order to identify the levels of IFN‐γ, TDLNs from mice with tumours were gathered and dissociated into single cells. Subsequently, tumour cell lysates were prepared by disrupting frozen tumour cells by three cycles of freeze–thaw cycles. These lysates were then utilised to activate tumour‐specific T cells. Following a 48‐h co‐incubation of the tumour lysates with lymph node cells, the supernatant was collected for analysis of IFN‐γ concentration using the mouse IFN‐γ ELISA kit (Proteintech, cat#KE10094).

### 2.7 Western blot

Cell lysates were obtained by using radioimmunoprecipitation assay buffer (RIPA) , and the protein concentration was quantified. The denatured proteins were then separated using sodium dodecyl sulfate polyacrylamide gel electrophoresis (SDS‐PAGE) gels with a gradient ranging from 6% to 15%, and subsequently transferred onto a polyvinylidene difluoride (PVDF) membrane. The PVDF membrane was blocked with 5% bovine serum albumin (BSA), and incubated with the indicated antibodies at 4°C. After thorough washing, the membrane was subjected to incubation with secondary antibodies conjugated with horseradish peroxidase (HRP). Finally, the protein bands were visualised. The primary antibodies were obtained from Cell Signalling Technology, Abcam or SantaCruz. The antibodies against phospho‐TBK1 (cat#5483), MyD88 (cat#4283), cyclic GMP‐AMP synthase (cGAS) (cat#31659), MAVS (cat#4983), STING (cat#13647), KDM5A (cat#3876), KDM4A (cat#ab191433), KDM4B (cat#8639), KDM4C (cat#sc515767), KDM4D (cat#sc393750), GAPDH (cat#2118), PD‐L1 (cat#ab213480), phospho‐histone H2A.X (Ser139) (cat#9718) and tri‐methyl‐histone H3 (Lys9) (cat#13969) were used.

### 2.8 Immunofluorescence analysis

To conduct immunofluorescence staining on cells, 50 000 tumour cells were seeded in glass bottom wells (Nunc, cat#150680) and incubated with JIB‐04 for 24 h. To visualise cytosolic DNA, live cells were incubated with PicoGreen (Thermo Fisher Scientific, cat#P11496) in serum‐free media. At the same time, 100 nM Mito‐tracker dye was employed to label the mitochondria. After staining the live cells, they were fixed for 10 min using 4% paraformaldehyde before being mounted with an anti‐fade medium containing 4',6‐diamidino‐2‐phenylindole (DAPI). In order to visualise HMGB1, fixed cells were washed, permeabilised, stained with specified antibodies at 4°C and then incubated with fluorescent secondary antibodies for 60 min at room temperature. Following the washing process, the nucleus was stained with DAPI. Pictures were obtained using confocal microscope, with a minimum of five images captured for each sample. The primary antibodies were obtained from Cell Signalling Technology, SantaCruz or Proteintech. The antibodies against HMGB1 (cat#3935), calreticulin (cat#12238), ERGIC‐53 (cat#sc‐365158), calnexin (cat#66903‐1‐Ig) and STING (cat#19851‐1‐AP) were used.

### 2.9 Flow cytometry

Tumour tissues were collected and subjected to mechanical dissociation, followed by digestion with a solution consisting of collagenase I, hyaluronidase and DNase I. To minimise nonspecific binding, cells were incubated with anti‐mouse CD16/32 antibody for 10 min, and then with Fixable Viability Stain for an additional 10 min to exclude non‐viable cells. After the washing process, cells were stained with specific antibodies for a duration of 30 min. To stain intracellular cytokines, tumour‐infiltrating leukocytes were isolated with an Optiprep (Sigma) density gradient, re‐stimulated for 5 h, and then fixed and permeabilised. Finally, cells were washed and analysed with the FACSAriaTM III flow cytometer (BD Biosciences). The primary antibodies were obtained from BD Biosciences or Biolegend. The antibodies used include Percpcy5.5‐CD45 (cat#103132), APC‐CD8 (cat#100712), BV605‐CD4 (cat#562658), BV421‐IFN‐γ (cat#505830), PE‐Tumor necrosis factor α (TNF‐α) (cat#506306) and Fixable Viability Stain 780 (cat#565388).

### 2.10 Detection of immunogenic cell death

After treatment with JIB‐04 for 24 h, cell supernatants were harvested to quantify the concentrations of HMGB1 (Elabscience, cat#E‐EL‐M0676c) and ATP (Beyotime Biotechnology, cat#S0026B) using enzyme‐linked immunosorbent assays (ELISA). To visualise HMGB1, immunofluorescence staining was carried out. Calreticulin expression was evaluated using flow cytometry.

### 2.11 Immunohistochemistry

Immunohistochemical staining (IHC) was performed on paraffin sections that had undergone deparaffinisation, rehydration and retrieval of tissue antigens. The sections were then stained with specific antibodies and subsequently treated with secondary antibodies labelled with fluorescein. Prior to analysing the images with a confocal microscope, nuclear staining was performed using DAPI, ensuring that a minimum of five images were obtained for each sample. The primary antibodies were obtained from Cell Signalling Technology. The antibodies against CD45 (cat#70257), CD8α (cat#44153), GranzymeB (cat#44153) and KDM4B (cat#8639) were used.

### 2.12 Cytosolic DNA extraction

To detect the levels of cytosolic DNA, cells were incubated with JIB‐04 for 24 h before being split into two equal aliquots. One aliquot was used to extract the total DNA, while the other was subjected to permeabilisation agents consisting of 50 μg/mL digitonin, 50 mM 4‐(2‐hydroxyethyl)‐1‐piperazineethanesulfonic acid (HEPES), 2 mM ethylenediaminetetraacetic acid (EDTA) and 150 mM NaCl. Following a 15‐min incubation, this mixture was centrifuged at 1000 *g* for 10 min, a step repeated thrice to collect supernatants. After centrifuging the supernatants at 17 000 *g* for 10 min, they were combined with an equivalent amount of ethanol (95%−100%) and then utilised for the extraction of cytosolic DNA. Both mtDNA and gDNA were then quantified using RT‐PCR.

### 2.13 Comet assay

To evaluate DNA damage, we utilised the neutral comet assay, which was conducted as previously described.^19^ The cells were diluted in PBS, mixed with .75% low melting point agarose, and then placed onto pre‐coated microscope slides until set. Afterwards, the slides were consecutively submerged in pre‐cooled lysis solution for 1 h at 4°C, then immersed in alkaline DNA unwinding solution at ambient temperature for 20 min. Post‐electrophoresis, the slides were DAPI stained and observed with a confocal microscope. The slides were completely air‐dried prior to image capture. One hundred randomly selected cells were scored per sample, and the extent of DNA migration was measured using the ‘OpenComet’ tool of ImageJ software.

### 2.14 Transcriptomic analysis

Following JIB‐04 treatment, total RNA was extracted. The cDNA libraries were generated with the NEBNext UltraTM RNA Library Prep Kit for Illumina (NEB), and then subjected to sequencing on the Illumina NovaSeq6000 system. The ‘DESeq2’ R package (version 4.2.3) was utilised to perform differential expression analysis between groups, with significant differential expression determined by thresholds of a corrected *p*‐value of .05 and absolute fold changes of 2. Gene set enrichment analysis was conducted on these differentially expressed genes using gene set enrichment analysis software (version 4.3.2).

### 2.15 Analysis of The Cancer Genome Atlas datasets

The RNA profiles and clinical data from The Cancer Genome Atlas (TCGA) were obtained from the UCSC Xena database (https://xenabrowser.net/datapages/). Immunotherapy data were obtained from the ROC Plotter database and Kaplan–Meier Plotter database. The TISIDB database was utilised to assess the immune cell infiltration of each TCGA tumour type.

### 2.16 IHC analysis of tissue microarray

Human tissue microarray of colon cancer, acquired from Shanghai Outdo Biotech Company, comprised 85 tumour tissues and 72 matched adjacent normal colon tissues. Clinical prognosis data for the patients were downloaded from the company's website. IHC staining was performed using antibodies against KDM4B (8639S; Cell Signalling Technology), and the intensity of KDM4B staining was quantified and scored. To ensure unbiased results, data collection was conducted in a double‐blinded manner.

### 2.17 Statistical analysis

Each experiment was independently carried out at least three times. The significance of differences between groups was determined via Student's *t*‐test, two‐way analysis of variance or log‐rank (Mantel–Cox) test. A *p*‐value < .05 was considered statistically significant.

## 3 RESULTS

### 3.1 Discovery of JIB‐04 as a novel epigenetic inhibitor for activating type I IFN signalling

**FIGURE 1:**
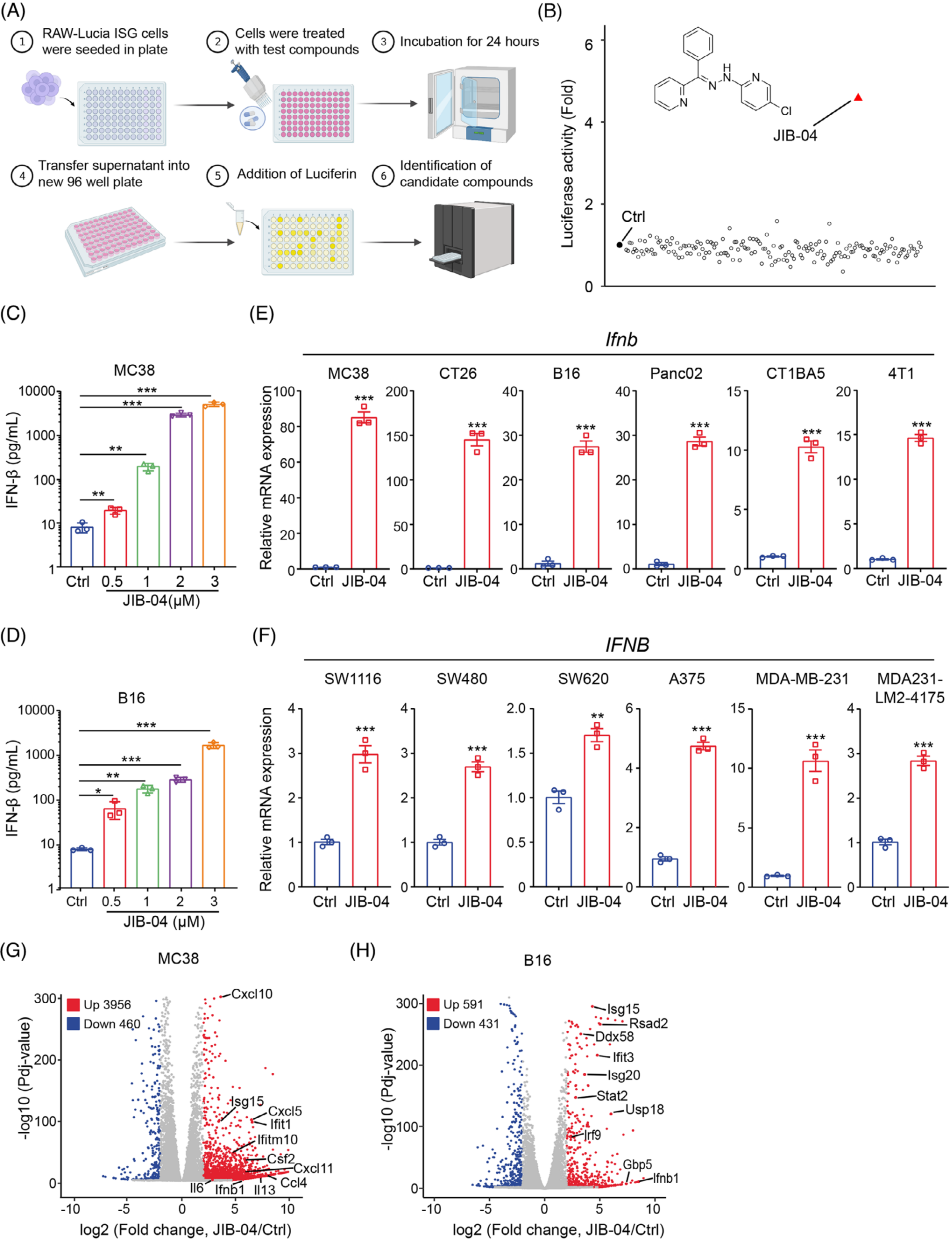
JIB‐04 efficiently stimulates type I interferons (IFNs) production in both human and mouse tumour cells. (A) Schematic overview of the high‐throughput drug screen workflow. RAW‐Lucia ISG cells were seeded in 96‐well plates and incubated with test compounds (1 μM) for 24 h. The supernatant was then transferred into a new 96‐well opaque white board, and QUANTI‐Luc substrate was added. The fluorescence intensity was immediately detected to assess the levels of type I IFN expression. (B) Drug screenings in cell models identify JIB‐04 as the most potent drug to trigger type I IFN production. JIB‐04 was highlighted in red and the chemical structure was shown. (C and D) MC38 (C) and B16 (D) cells were treated with indicated dose of JIB‐04 for 24 h and the culture supernatants were collected for IFN‐β protein detection by enzyme‐linked immunosorbent assays (ELISA). (E and F) Indicated mouse (E) and human (F) tumour cells were treated with JIB‐04 (2 μM) for 24 h, and cells were collected for IFN‐β mRNA detection by quantitative real‐time polymerase chain reaction (RT‐qPCR). (G and H) Volcano plots showing the differentially expressed genes between the control and JIB‐04‐treated MC38 (G) or B16 (H) cells. The downregulated genes were represented in blue, and the upregulated genes were represented in red. Several type I IFN‐related genes were highlighted in red on the plot. Data are shown as mean ± SEM (*n* = 3). *p*‐Value was calculated by unpaired Student's *t*‐test (^*^ *p* < .05, ^**^ *p* < .01, ^***^ *p* < .001).

To identify novel epigenetic regulators that control tumour response to host immunity, we employed a curated RAW‐Lucia ISG cell‐based screening^20^ with epigenetic inhibitor compounds. The activation of type I IFNs was used as a readout, given its known role in regulating tumour responses to host immunity (Figure 1A). A total of 176 compounds were included in this Epigenetics Inhibitor Compounds Library (Table S3). Among the compounds investigated, JIB‐04 emerged as the most effective in inducing type I IFN production (Figure 1B). JIB‐04 is a small molecular inhibitor of multiple members of KDM 4, 5 and 6 families, and has shown potential therapeutic value in treating solid tumours.^21^ We confirmed the enhanced production of IFN‐β at both protein and mRNA levels in various cancer cell lines, including colorectal (MC38 and CT26), melanoma (B16), pancreatic (Panc02 and CT1BA5) and breast (4T1) cancer cells. ELISA results revealed that JIB‐04 dose dependently induced the production of IFN‐β, with a 100‐fold increase observed at a concentration of 3 μM (Figure 1C,D). Consistently, IFN‐β expression was significantly increased across all tested tumour cells after JIB‐04 treatment (Figure 1E), suggesting that JIB‐04 acts on the transcriptional regulation to promote protein production. We further validated the stimulatory impact of JIB‐04 on IFN‐β expression in human colorectal, melanoma and breast cancer cells (Figure 1F). This transcriptional effect was also concentration‐dependent (Figure S1A). Moreover, we observed a similar effect of JIB‐04 on the expression of the downstream signalling genes (Cxcl10, Isg15 and antigen presentation‐related genes) of IFN‐β in both human and mouse cancer cells (Figure S1B–D). Transcriptomic analysis further showed a notable enrichment of type I IFN pathway in MC38 and B16 cells after JIB‐04 treatment (Figures 1G,H and S1E), providing additional evidence of type I IFN signalling activation. Collectively, these results demonstrate that JIB‐04 is an effective epigenetic drug capable of triggering robust tumour‐intrinsic type I IFN signalling.

### 3.2 JIB‐04 triggers tumour‐intrinsic cytosolic DNA sensing via cGAS/STING pathway

**FIGURE 2:**
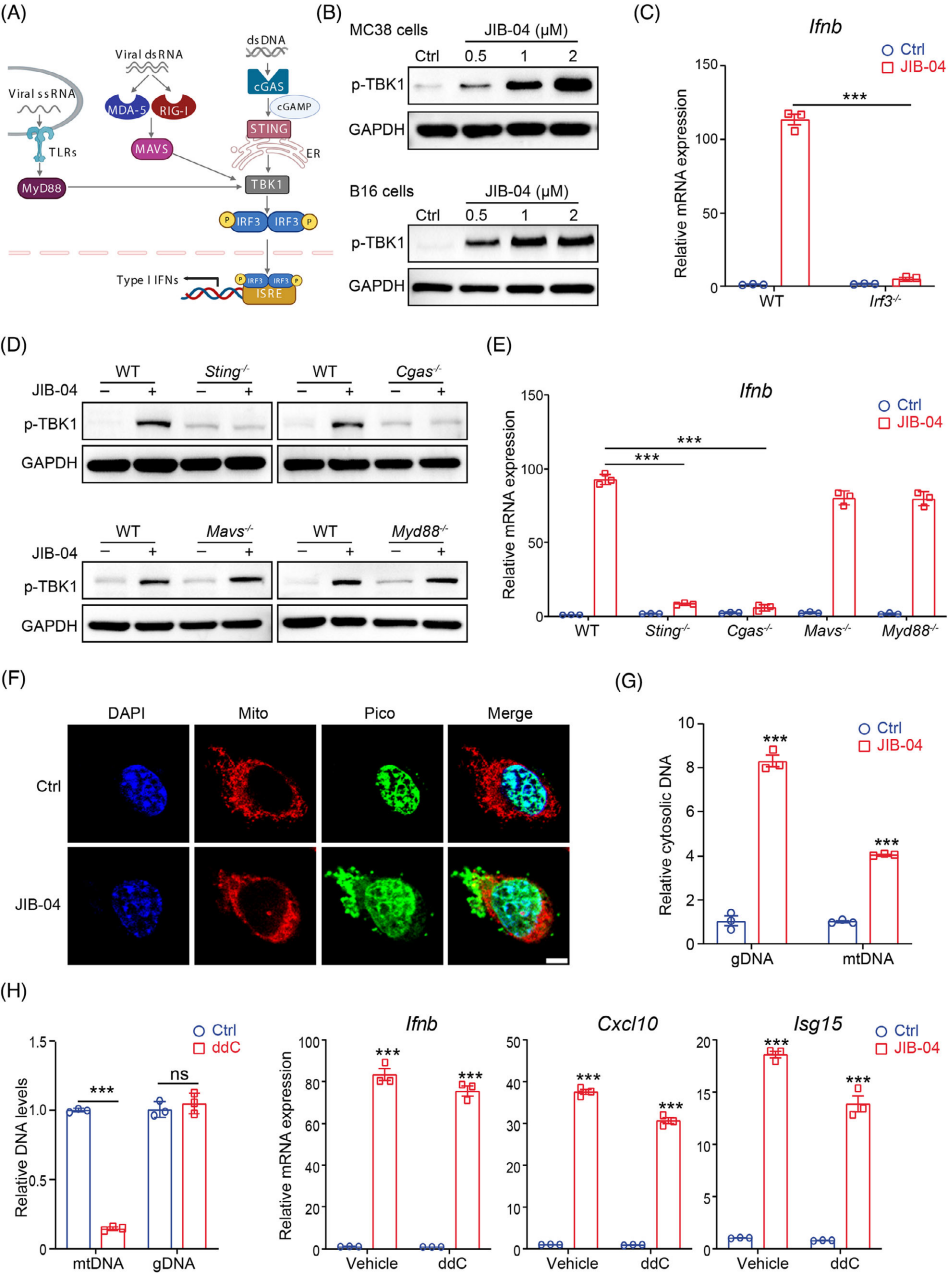
JIB‐04 promotes the release of tumour‐intrinsic cytosolic DNA and activates DNA sensing via the cyclic GMP‐AMP synthase (cGAS)/STING pathway. (A) Schematic diagram of the main pathways that stimulate the production of type I interferons (IFNs). (B) MC38 (top) and B16 (bottom) cells were treated with indicated dose of JIB‐04 for 24 h, and the protein level of phosphorylated TBK1 (p‐TBK1) was determined by western blot. (C) Wild‐type (WT) and Irf3‐deficient MC38 cells were treated with 2 μM JIB‐04 for 24 h, and the mRNA level of IFN‐β was determined by quantitative real‐time polymerase chain reaction (RT‐qPCR). (D and E) MC38 cells with indicated gene knockout were treated with 2 μM JIB‐04 for 24 h. The protein level of p‐TBK1 (D) was determined by western blot; the mRNA level of IFN‐β (E) was determined by RT‐qPCR. (F) Representative confocal images showing cytosolic DNA (Picogreen, green), mitochondria (Mito‐Tracker, red) and nuclei (DAPI, blue) in MC38 cells after JIB‐04 treatment (2 μM, 24 h). Scale bar, 5 μm. (G) MC38 cells were treated with 2 μM JIB‐04 for 24 h, and the levels of gDNA and mtDNA were determined by RT‐qPCR. (H) MC38 cells were treated with dideoxycytidine (ddC, 50 μM) for 10 days to deplete mtDNA. The deletion efficiency was verified and the expression of IFN‐β, Cxcl10 and Isg15 was determined by RT‐qPCR. Data are shown as mean ± SEM (*n* = 3). *p*‐Value was calculated by unpaired Student's *t*‐test (^***^ *p* < .001).

To explore the mechanisms responsible for the activation of type I IFNs by JIB‐04, we first focused on the impact of JIB‐04 on the crucial steps involved in the type I IFN production. Different sensors, including cGAS, toll‐like receptors and retinoic acid‐inducible gene I‐like receptors, can induce cell‐intrinsic type I IFNs through recognition of nucleic acids. When cytosolic nucleic acids are detected, these sensors initiate signalling cascades that converge on the activation of TANK‐binding kinase 1 (TBK1) and interferon regulatory factor 3 (IRF3), ultimately resulting in the production of type I IFNs (Figure 2A). Firstly, we analysed the expression of the signalling hub downstream, and found that JIB‐04 markedly activated TBK1 in a dose‐dependent manner in MC38 and B16 cells (Figure 2B). Importantly, loss of IRF3 eliminated the enhancing effect of JIB‐04 on IFN‐β expression, as well as downstream genes like Cxcl10 and Isg15 (Figures 2C andS2A,B), thereby validating the key role of TBK1/IRF3 axis in this process. Next, to determine the upstream pathway responsible for JIB‐04‐induced type I IFN production, we performed targeted knockouts of key mediators within each signalling pathway (Figure S2A). Similar to wild type, deletion of MyD88 or MAVS had negligible impact on TBK1 activation and the expression of IFN‐β and downstream genes (Figures 2D,E and S2C). However, deletion of cGAS or STING manifested the same outcomes as the deletion of IRF3 (Figures 2D,E and S2C), highlighting a crucial role of the cGAS/STING pathway in this signalling cascade. Furthermore, we validated the activation of STING signalling via immunofluorescence. Typically, activated STING relocates from the endoplasmic reticulum (ER) to the ER‐Golgi intermediate compartment (ERGIC), where it engages TBK1 and IRF3 to initiate type I IFN expression. Following JIB‐04 treatment, we observed an increased STING accumulation within the ERGIC and reduced STING distribution on the ER (Figure S2D), indicating that JIB‐04 facilitates STING trafficking and triggers type I IFN production.

Besides sensing exogenous DNA from pathogens, dsDNA sensor cGAS can be activated by endogenous cytosolic DNA.^22^ Accordingly, we examined the change in cytosolic dsDNA by staining cells with Picogreen, and observed a significant accumulation of cytosolic DNA after treatment of JIB‐04 (Figures 2F and S3A). Endogenous cytosolic DNA is mainly derived from mitochondria (mtDNA) and nuclear DNA leakage (gDNA). Indeed, JIB‐04 significantly enhanced the concentrations of both gDNA and mtDNA in the cytoplasm (Figures 2G and S3B). To investigate the relative contributions of mtDNA and gDNA in activating type I IFNs, we used dideoxycytidine to selectively deplete mtDNA (Figure 2H). It is important to mention that even though mtDNA was depleted, the expression of type I IFNs and downstream signalling genes was only partially diminished, rather than completely eliminated (Figure 2H). This observation strongly suggests that while both mtDNA and gDNA contribute to the activation of type I IFNs induced by JIB‐04, gDNA seems to be the more predominant factor in this mechanism. Collectively, these results demonstrate that JIB‐04 induces the release of endogenous cytosolic DNA, initiates cGAS/STING sensing pathway and subsequently promotes the production of type I IFNs.

### 3.3 JIB‐04 induces DNA damage and triggers an immunogenic cell death

**FIGURE 3:**
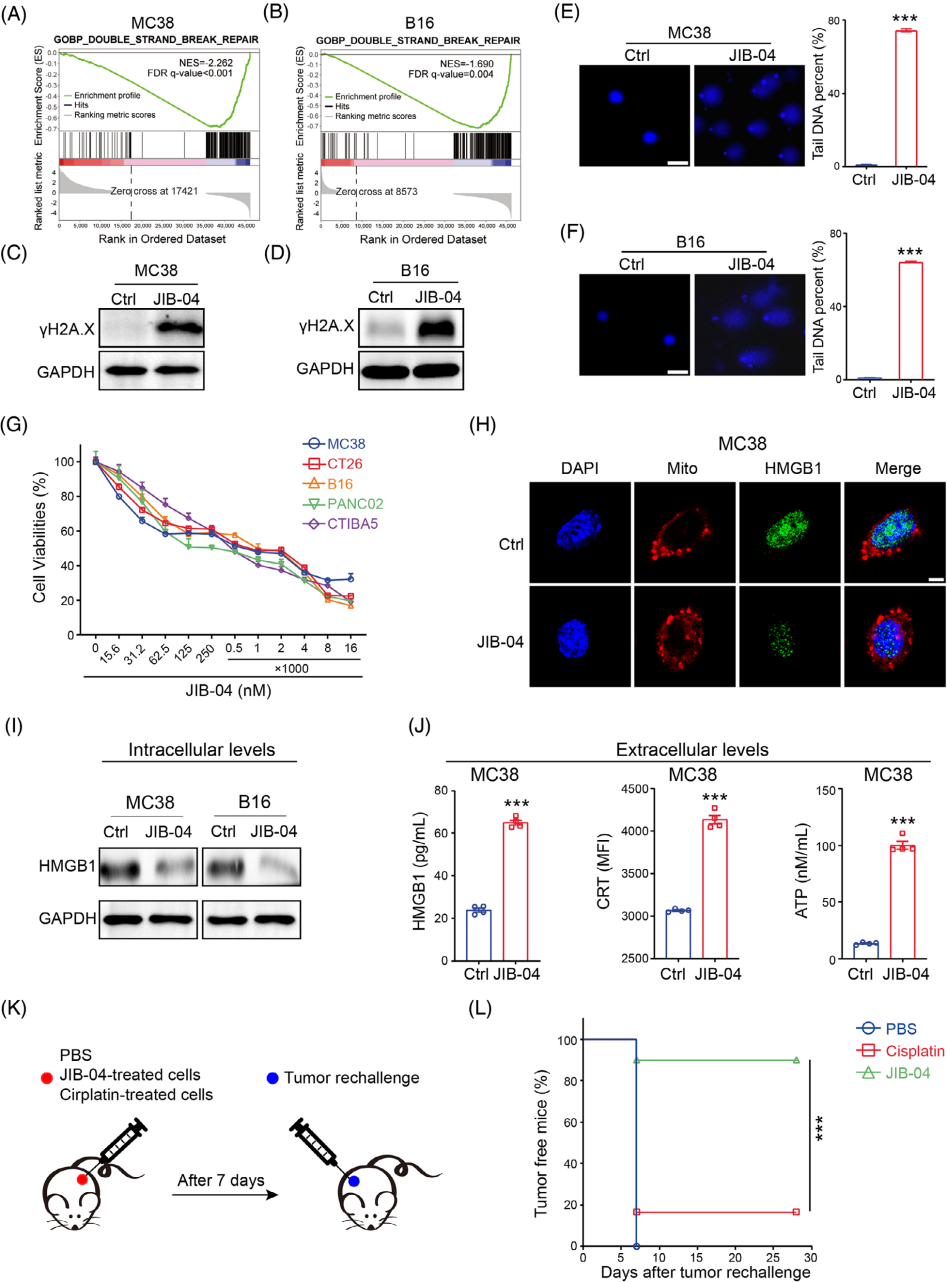
JIB‐04 triggers DNA damage and immunogenic cell death of tumour cells. (A and B) Gene set enrichment analysis of differential expressed genes between the control and JIB‐04‐treated MC38 (A) or B16 (B) cells. (C and D) MC38 (C) and B16 (D) cells were treated with 2 μM JIB‐04 for 24 h, and the protein level of γH2A.X was determined by western blot. (E and F) Representative images and quantification of DNA comet assays in MC38 (E) and B16 (F) cells with JIB‐04 treatment. More than 100 cells were analysed in every group. Scale bar, 50 μm. (G) MC38, CT26, B16, Panc02 and CTIBA5 cells were treated with JIB‐04 at various concentrations for 24 h, and cell viability was determined by Cell Counting Kit‐8 (CCK‐8) assay. (H) Representative confocal images showing HMGB1 (green), mitochondria (red) and nuclei (blue) in MC38 cells after JIB‐04 treatment. Scale bar, 5 μm. (I) The intracellular protein levels of HMGB1 in MC38 and B16 cells were determined by western blot after JIB‐04 treatment. (J) The extracellular levels of HMGB1 (left) and ATP (right) were detected by enzyme‐linked immunosorbent assays (ELISA), and the extracellular expression of calreticulin (CRT) (middle) was detected by flow cytometry (*n* = 4). (K) Scheme of in vivo tumour vaccination‐rechallenge model. Six‐week‐old female C57BL/6 mice were divided into three groups (phosphate‐buffered saline [PBS], *n* = 10; JIB‐04, *n* = 10; cisplatin, *n* = 12) and subcutaneously inoculated with MC38 cells treated with PBS, lethal doses of cisplatin or JIB‐04, respectively. After 7 days, live MC38 cells were injected into the contralateral flank of mice. (L) The percentage of tumour‐free mice (tumour volume less than 100 mm^3^) was shown. Data are shown as mean ± SEM. *p*‐Value was calculated by unpaired Student's *t*‐test in (E), (F) and (J) or log‐rank test in (L) (^***^ *p* < .001).

The leakage of nuclear DNA indicates DNA damage and subsequent cell death. Therefore, we reasoned that JIB‐04 would be able to cause DNA damage. The major cause of DNA damage accumulation is the malfunction of DNA repair pathways.^23^ According to our RNA‐seq analysis, JIB‐04 treatment specifically impaired DNA double‐strand break (DSB) repair pathways (Figure 3A,B). To determine the formation and extent of DSB in response to JIB‐04, we examined the levels of phosphorylated histone H2AX (γH2AX), a known hallmark of DNA DSB.^24^ As expected, the expression of γH2A.X was notably enhanced in both MC38 and B16 cells following JIB‐04 treatment (Figure 3C,D). Additionally, using the alkaline comet assay, we observed a substantial increase in comet tails in JIB‐04‐treated cells (Figure 3E,F), further validating the occurrence of DNA DSB. Consequently, the accumulation of unrepaired DNA DSB triggered a cell death response, as evidenced by the concentration‐dependent loss of cell viability in JIB‐04‐treated cells (Figure 3G).

ICD has recently gained considerable attention as a pivotal factor in the development of therapy‐induced antitumour immunity.^25^ To determine whether JIB‐04 is capable of inducing ICD in tumour cells, we examined the basic molecular processes of ICD. These processes include the release of HMGB1 as a ‘find me’ signal, the externalisation of calreticulin (CRT) on the cell membrane as an ‘eat me’ signal, as well as the secretion of ATP.^26^ Our immunofluorescence staining revealed that treatment of JIB‐04 led to a greater extracellular release of HMGB1 (Figure 3H). Additionally, both intracellular HMGB1 expression and the extracellular HMGB1 levels of JIB‐04‐treated cells showed similar results (Figure 3I,J), indicating that a considerable amount of HMGB1 was released into the extracellular space. Likewise, the cell surface expression of CRT and the secretion of ATP further validated the ability of JIB‐04 to induce ICD (Figure 3J). To confirm whether JIB‐04 can induce immunogenicity in vivo, we performed a tumour vaccination experiment. As a vaccine, dying tumour cells induced in vitro by either JIB‐04 or cisplatin (an inefficient ICD inducer), were injected into the flank of mice. Seven days post‐vaccination, we rechallenged mice with live tumour cells on the contralateral side (Figure 3K). Immunisation with JIB‐04‐treated cells inhibited the growth of the rechallenged tumour with a 90% tumour‐free survival rate (Figure 3L). In contrast, mice immunised with dying cells induced by cisplatin failed to show any significant reduction in rechallenged tumour growth (Figure 3L). Collectively, these results indicate that JIB‐04 promotes the leakage of nuclear DNA by inducing DNA damage, which subsequently induces ICD and enhances specific antitumour immunogenicity.

### 3.4 JIB‐04 activates tumour‐intrinsic innate sensing by inhibiting KDM4 family

JIB‐04 functions a potent inhibitor of Jumonji domain demethylases that effectively inhibits KDM4 family, KDM5A and KDM6B with high potency at nanomolar concentrations.^21^ To investigate whether JIB‐04‐mediated innate sensing arises from the inhibition of its target genes, we selectively inhibited each target gene using genetic knockout or small molecule inhibitor. It is noteworthy that neither KDM5A knockout nor KDM6B inhibition via small molecule inhibitor GSK‐J1 was able to induce TBK1 activation and IFN‐β production (Figure S4A–C), thereby ruling out the involvement of KDM5A and KDM6B in this mechanism. In contrast, TACH101, a clinical trial‐phase inhibitor specifically targeting the KDM4 family, markedly increased IFN‐β expression and induced ICD (Figure S4D–F). This distinct response suggests that JIB‐04's primary mechanism of action predominantly involves the inhibition of the KDM4 family.

**FIGURE 4:**
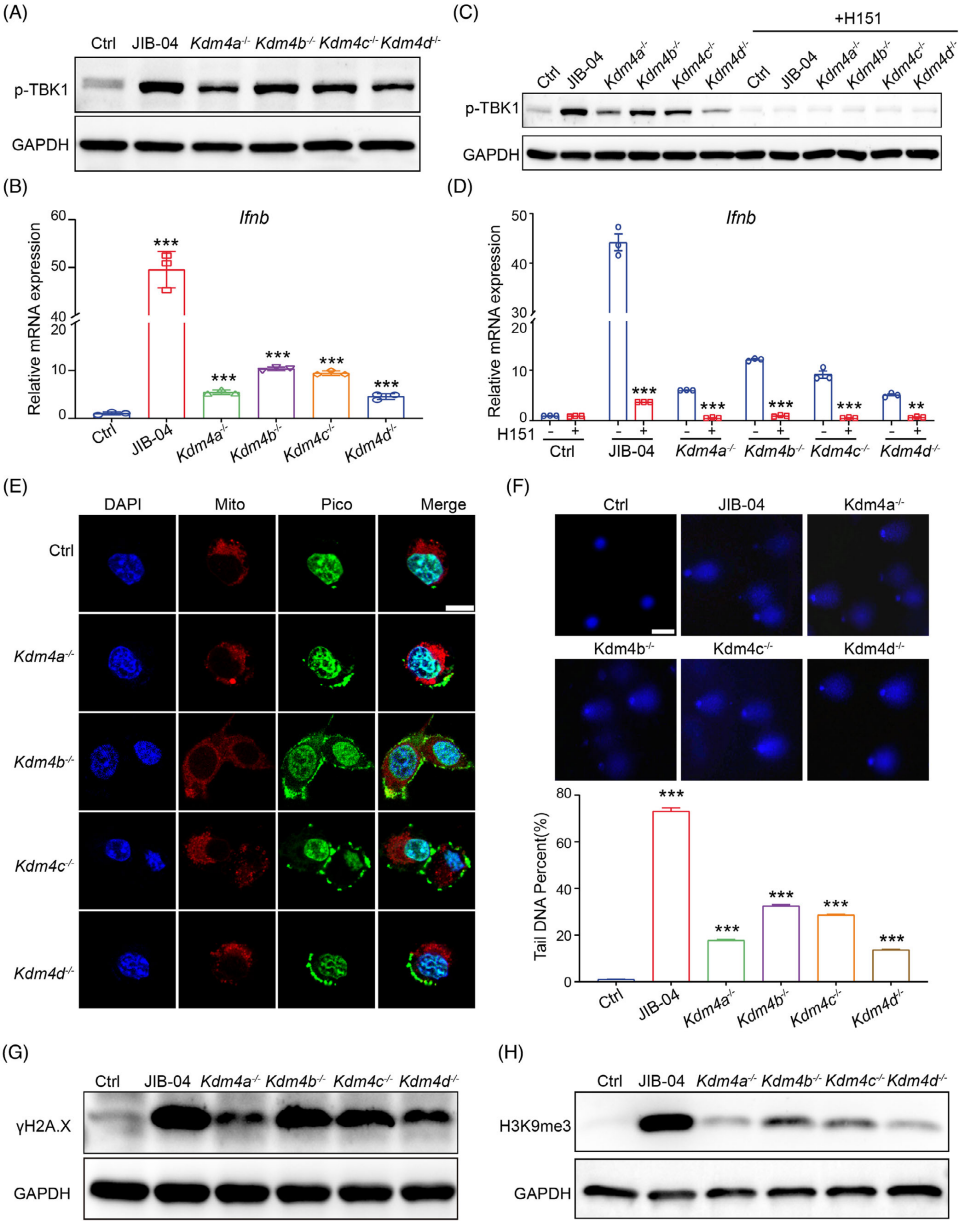
JIB‐04 inhibits KDM4 family to promote DNA damage and activate DNA sensing. (A and B) Indicated genes were knocked out in MC38 cells, and the protein level of p‐TBK1 (A) was determined by western blot; the mRNA level of interferon (IFN)‐β (B) was determined by quantitative real‐time polymerase chain reaction (RT‐qPCR) (*n* = 3). JIB‐04 treatment was used as a positive control. (C and D) MC38 cells with indicated gene knockout were treated with STING inhibitor (H151), and the protein level of p‐TBK1 (C) was determined by western blot; the mRNA level of IFN‐β (D) was determined by RT‐qPCR (*n* = 3). (E) Representative confocal images showing cytosolic dsDNA (green), mitochondria (red) and nuclei (blue) in MC38 cells with indicated gene knockout. Scale bar, 10 μm. (F) Representative images (top) and quantification (bottom) of DNA comet assays in MC38 cells with indicated gene knockout. More than 100 cells were analysed in every group. Scale bar, 50 μm. (G and H) The protein levels of γH2A.X (G) and H3K9me3 (H) in MC38 cells with indicated gene knockout were determined by western blot. Data are shown as mean ± SEM. *p*‐Value was calculated by unpaired Student's *t*‐test (^***^ *p* < .001).

The murine KDM4 family, consisting of KDM4A, KDM4B, KDM4C and KDM4D, has a specific role in catalysing the removal of methyl groups from histone H3 lysine 9 (H3K9). To determine their ability to activate type I IFNs, we individually knocked out each KDM4 family member in MC38 and B16 cells (Figures S4A and S5A). We found that deleting each KDM4 family gene induced TBK1 activation and IFN‐β production (Figures 4A,B and S5B,C). However, their activation ability was notably inferior to that of JIB‐04, strongly indicating that JIB‐04‐induced type I IFN activation originates from the suppression of multiple KDM4 family members, rather than solely via the inhibition of an individual gene. Among the four members, KDM4B knockout showed the highest capacity to stimulate type I IFN production among the four members, whereas the effects of the remaining members were comparatively less potent (Figures 4B and S5C). Furthermore, the stimulatory effects of type I IFN production induced by genetic deletion of KDM4 were also dependent on the STING pathway, as demonstrated by the complete abolishment of effects upon treatment with a STING inhibitor (H151) (Figure 4C,D).

Next, we evaluated whether KDM4 deletion would induce the release of cytosolic DNA and DNA damage, as observed with JIB‐04 treatment. As expected, the deletion of each KDM4 member promoted the accumulation of cytosolic DNA (Figures 4E and S5D). Similar to JIB‐04, the deletion of each KDM4 member also significantly increased the comet tails and the expression of γH2AX (Figures 4F,G and S5E,F). The degree of DNA damage induced by KDM4 deletion was directly correlated with the production of IFN‐β. Previous research has indicated that aberrant hypermethylation of H3K9 across the entire genome impedes the gathering of DNA repair elements at DNA breaks, resulting in impaired DNA repair and causing DNA damage.^27^ In our study, we found that the deletion of each KDM4 family member or treatment with JIB‐04 resulted in H3K9 hypermethylation, thereby elucidating a mechanism by which JIB‐04 induces DNA damage (Figures 4H and S5G).

In general, members belonging to the same subfamily show the similar expression patterns. Indeed, we noted a robust correlation in the expression of KDM4A, KDM4B, KDM4C and KDM4D across all tumour samples in the TCGA pan‐cancer database (Figure S6). The co‐expression patterns imply the presence of functional redundancy among these genes, indicating that the inhibition of a single gene does not exert the same inhibitory effect as the simultaneous inhibition of all four genes. Taken together, these findings suggest that JIB‐04, through inhibiting KDM4 family genes, induces DNA damage, activates innate sensing and promotes the synthesis of type I IFNs.

### 3.5 Targeting KDM4 family activates innate and adaptive antitumour immunity

**FIGURE 5:**
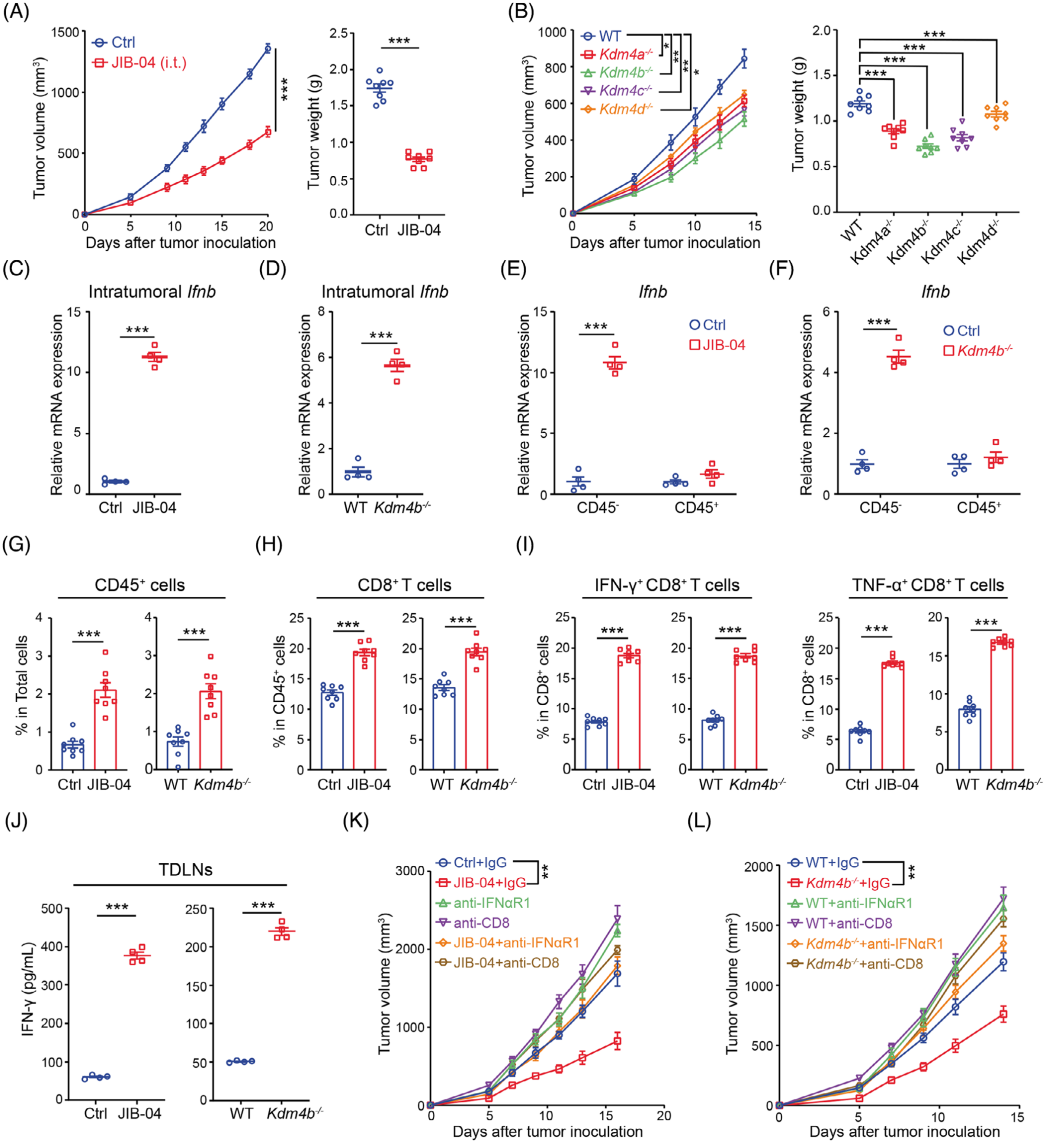
Targeting KDM4 family inhibits tumour growth by activating type I interferon (IFN) signalling and T‐cell immunity. (A) C57BL/6 mice (*n* = 8 per group) were inoculated with 1 × 10^6^ MC38 cells and subjected to intratumoural treatment with JIB‐04. Tumour growth (left) and weights (right) were measured. (B) C57BL/6 mice (*n* = 8 per group) were inoculated with 1 × 10^6^ wild‐type (WT) or Kdm4a/b/c/d‐deficient MC38 cells. Tumour growth (left) and weights (right) were measured. (C and D) The mRNA expression of IFN‐β in JIB‐04‐treated (C) or Kdm4b‐deficient (D) tumour tissues was determined by quantitative real‐time polymerase chain reaction (RT‐qPCR). (E and F) The mRNA expression of IFN‐β in live tumour cells (CD45−) and immune cells (CD45+) sorted from JIB‐04‐treated (E) or Kdm4b‐deficient (F) tumour tissues was determined by RT‐qPCR. (G–I) The proportion of infiltrating CD45+ cells (G), CD8+ T cells (H), IFN‐γ+ CD8+ T cell (I), and TNF‐α+ CD8+ T cell (I) in the tumour microenvironment (TME) of MC38 tumour‐bearing mice was detected by flow cytometry. (J) The tumour‐draining lymph nodes (TDLNs) of MC38 tumour‐bearing mice were dissected, and then dissolved into suspensions. After re‐stimulated with dead MC38 cells for 48 h, the supernatant was collected for IFN‐γ detection by enzyme‐linked immunosorbent assays (ELISA). (K) MC38 tumour‐bearing mice (*n* = 8 per group) were treated with JIB‐04, anti‐IFNαR1 or anti‐CD8 antibodies. Tumour growth were measured. (L) WT or Kdm4b‐deficient MC38 tumour‐bearing mice (*n* = 8 per group) were treated with anti‐IFNαR1 or anti‐CD8 antibodies. Tumour growth were measured. Data are shown as mean ± SEM. *p*‐Value was calculated by unpaired Student's *t*‐test in (C–J) or two‐way analysis of variance (ANOVA) in (A), (B), (K) and (L) (^**^ *p* < .01, ^***^ *p* < .001).

We next evaluated the antitumour effects of KDM4 inhibition in syngeneic tumour models. To minimise potential off‐target side effects of JIB‐04, JIB‐04 was intratumourally administrated to tumour‐bearing mice following tumour inoculation. The antitumour potency of JIB‐04 was quantified at varied daily doses (.19, .57 and .95 mg per injection). Impressively, all the doses significantly inhibited tumour growth in comparison to the control group, with the .57 and .95 mg doses demonstrating particularly potent effects (Figure S7A). Given their comparable efficacy, we selected the dose of .57 mg per injection for subsequent studies. We also investigated the systemic therapeutic effects of JIB‐04 through intraperitoneal administration. Notably, intratumoural delivery, which achieved an approximately 50% tumour inhibition rate (TIR) (Figures 5A and S7B), was more effective than intraperitoneal administration, which yielded a comparatively modest TIR of around 25% (Figure S7C). Additionally, no obvious differences in mouse body weight were observed between the JIB‐04 treatment and control group (Figure S7D), implying both efficient and safe actions. Similar to JIB‐04 treatment, genetic deletion of KDM4 also resulted in marked tumour growth inhibition (Figure 5B). Of note, KDM4B deletion yielded the most robust antitumour effect across all KDM4 family members (Figure 5B), aligning with its impact on type I IFN production (Figure 4).

Our subsequent inquiry focused on determining if JIB‐04 could enhance the generation of type I IFNs within the TME. Compared to control group, treatment of JIB‐04 or deletion of KDM4B markedly elevated intratumoural IFN‐β mRNA levels (Figures 5C,D and S8A). In order to further identify the source of increased IFN‐β in vivo, we isolated tumour cells (CD45^−^) and immune cells (CD45^+^) from tumour tissues and compared the expression of IFN‐β between the two cell populations. Our findings indicate that it is primarily tumour cells, not immune cells, contribute to the enhanced production of IFN‐β after JIB‐04 treatment or KDM4B deletion (Figure 5E,F).

T‐cell infiltration and activation are important aspects of the adaptive immune response against tumour progression. In order to investigate whether KDM4 inhibition activated the adaptive immunity, we utilised flow cytometry to assess the immune makeup in the TME, with a focus on T cells. Treatment of JIB‐04 or deletion of KDM4B led to greater immune cell infiltration, particularly CD8^+^ T cells, and enhanced their cytotoxic function (IFN‐γ and TNF‐α) within the TME compared to control group (Figures 5G–I and S8B,C). The findings were further supported by IHC analysis, which showed that both pharmacological and genetic inhibition of KDM4 greatly enhanced the infiltration and activation of CD8^+^ T cells (as indicated by Granzyme‐B) (Figure S8D). Furthermore, we observed that deletion of KDM4B among all KDM4 family members yielded the greatest amount of Granzyme‐B in tumour tissues (Figure S8D). Importantly, the genetical inhibition‐induced activation of CD8^+^ T cells exhibited a positive correlation with the observed therapeutic outcome, suggesting the pivotal role of CD8^+^ T cells in this process. To further analyse therapy‐induced T‐cell activation, we performed an in vitro co‐culture assay to evaluate the tumour‐specific reactivity of T cells. Specifically, lymphocytes from extracted TDLNs were cultured with autologous‐tumour cells, with IFN‐γ production serving as a measure of tumour reactivity. In comparison to the control group, JIB‐04 markedly enhanced the tumour‐specific T‐cell response (Figures 5J and S8E). Importantly, the efficacy of JIB‐04 in enhancing IFN‐β expression and T‐cell infiltration, as well as its antitumour activity were largely diminished in STING‐deficient tumours (Figure S8F,G), highlighting the essential role of STING/type I IFN axis in reshaping the T‐cell‐inflamed TME.

To assess if local administration of JIB‐04 could elicit systemic antitumour effects, we conducted experiments on mice with bilateral subcutaneous MC38 or B16 tumours. Tumour cells were implanted subcutaneously on both flanks, with only the left tumour (primary) receiving intratumoural injections of JIB‐04, while the right tumour (distant) was left untreated. The results demonstrated that JIB‐04 markedly delayed the growth of both tumours in the MC38 and B16 tumour models (Figure S9). Intriguingly, the distant tumours exhibited a larger size compared to the primary tumours (Figure S9). This suggests that, besides its inherent cytotoxicity, JIB‐04 may elicit more robust immune responses at the treatment site, potentially due to the inhibition of KDM4.

To address the impact of innate and adaptive immune responses in the antitumour outcome, we employed specific interventions: neutralising type I IFN signalling with an anti‐IFNαR1 antibody, and depleting CD8^+^ T cells with an anti‐CD8 antibody or utilising T‐cell‐deficient nude mice as a model. Remarkably, the therapeutic effect of JIB‐04 treatment and KDM4B deletion was greatly diminished by blocking type I IFN signalling or depleting CD8^+^ T cells (Figure 5K,L). Furthermore, the administration of JIB‐04 in nude mice also exhibited a markedly reduced therapeutic response compared to that in immunocompetent mice (Figure S10). In conclusion, these results demonstrate that innate and adaptive immunity essentially mediate the antitumour effects induced by KDM4 inhibition.

### 3.6 Targeting KDM4 family synergises with immunotherapy to promote antitumour immunity

**FIGURE 6:**
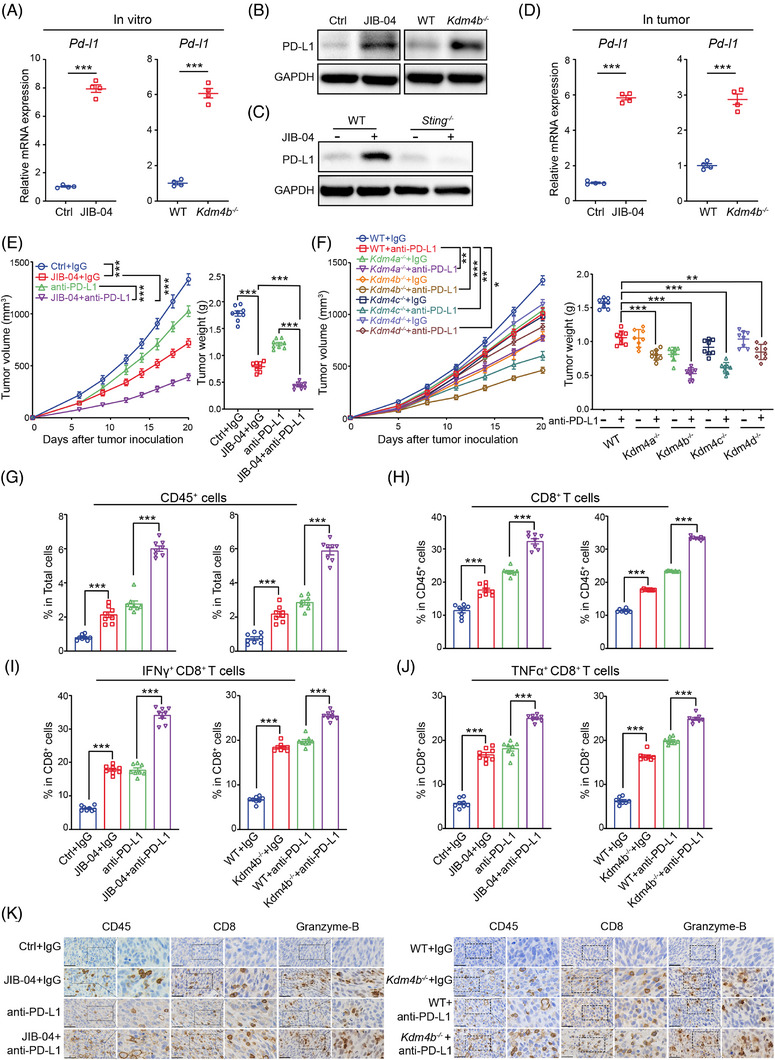
FIGURE 6 Targeting KDM4 family synergises with programmed death‐ligand 1 (PD‐L1) blockade to overcome adaptive immune resistance and enhance antitumour effects. (A and B) JIB‐04‐treated (left) and Kdm4b‐deficient (right) MC38 cells were collected for PD‐L1 mRNA (A) and protein (B) detection (*n* = 4). (C) Wild‐type (WT) and Sting‐deficient MC38 cells were treated with JIB‐04 for 24 h, and the protein level of PD‐L1 was determined by western blot. (D) The mRNA expression of PD‐L1 in the JIB‐04‐treated (left) and Kdm4b‐deficient (right) tumour tissues were determined by quantitative real‐time polymerase chain reaction (RT‐qPCR) (*n* = 4). (E) C57BL/6 mice (*n* = 8/group) were inoculated with 1 × 10^6^ MC38 cells and subjected to JIB‐04 treatment and/or anti‐PD‐L1 antibodies. Tumour growth (left) and weights (right) were measured. (F) C57BL/6 mice (*n* = 8/group) were inoculated with 1 × 10^6^ WT or Kdm4a/b/c/d‐deficient MC38 cells and/or subjected to anti‐PD‐L1 antibodies. Tumour growth (left) and weights (right) were measured. (G–J) The proportion of infiltrating CD45+ cells (G), CD8+ T cells (H), interferon (IFN)‐γ+ CD8+ T cell (I) and TNF‐α+CD8+ T cell (J) in the tumour microenvironment (TME) after indicated treatments was detected by flow cytometry. (K) Representative immunohistochemistry images showing the proportion of tumour‐infiltrating immune cells and CD8+ T cells as well as the expression of Granzyme B in MC38 tumour tissues after indicated treatments. Scale bar, 40 μm. Data are shown as mean ± SEM. *p*‐Value was calculated by unpaired Student's *t*‐test in (A), (D–J) or two‐way analysis of variance (ANOVA) in (E) and (F) (^**^ *p* < .01, ^***^ *p* < .001).

The interaction between PD‐L1 on tumour cells and PD‐1 on T cells plays a crucial role in controlling the immune response against tumour cells, resulting in T‐cell exhaustion and immune evasion.^28^ Previous studies indicate that type I IFNs are among the most potent inducers of PD‐L1, which dampen the T‐cell response.^29^, ^30^ In order to investigate the potential involvement of the PD‐1/PD‐L1 pathway in adaptive immune resistance after KDM4 inhibition, we measured PD‐L1 levels on tumour cells following JIB‐04 treatment or genetic deletion of KDM4B. Our results revealed that both JIB‐04 treatment or KDM4B deletion significantly increased PD‐L1 expression (Figure 6A,B). This increased PD‐L1 expression was predominantly reliant on tumour‐intrinsic type I IFN signalling, as the absence of STING almost eliminated the induction of PD‐L1 by JIB‐04 treatment (Figure 6C). Moreover, an enhanced expression of PD‐L1 was detected in JIB‐04‐treated or KDM4B‐deficient tumour tissues (Figure 6D).

To counteract the adverse effects of the upregulated PD‐L1 on antitumour immune response, we treated mice with anti‐PD‐L1 antibodies. Given the susceptibility of MC38 cells to anti‐PD‐1/PD‐L1 immunotherapy, we reduced the dose to 50 μg anti‐PD‐L1 antibodies per mouse every 4 days. We observed that PD‐L1 blockade alone modestly inhibited MC38 tumour growth in our experimental settings, while combination with JIB‐04 treatment or KDM4 deletion produced a stronger antitumour effect (Figure 6E,F). Similarly, this combination strategy also enhanced the sensitivity of resistant B16 tumours to PD‐L1 blockade (Figure S11A). Among all KDM4 family members, KDM4B deletion exhibited the strongest synergistic effect with PD‐L1 blockade, resulting in a TIR of up to 75% (Figure 6F). This impressive synergy was associated with an increased infiltration and activation of immune cells, particularly CD8^+^ T cells, in the tumour tissues, suggesting a successful reactivation of T‐cell immunity (Figures 6G–K and S11B,C). Above all, our results demonstrate that targeting KDM4 evolves adaptive immune resistance through the increase in PD‐L1 expression, which can be effectively countered by blocking PD‐L1.

### 3.7 KDM4B expression is negatively correlated with type I IFN signature and clinical outcomes

**FIGURE 7:**
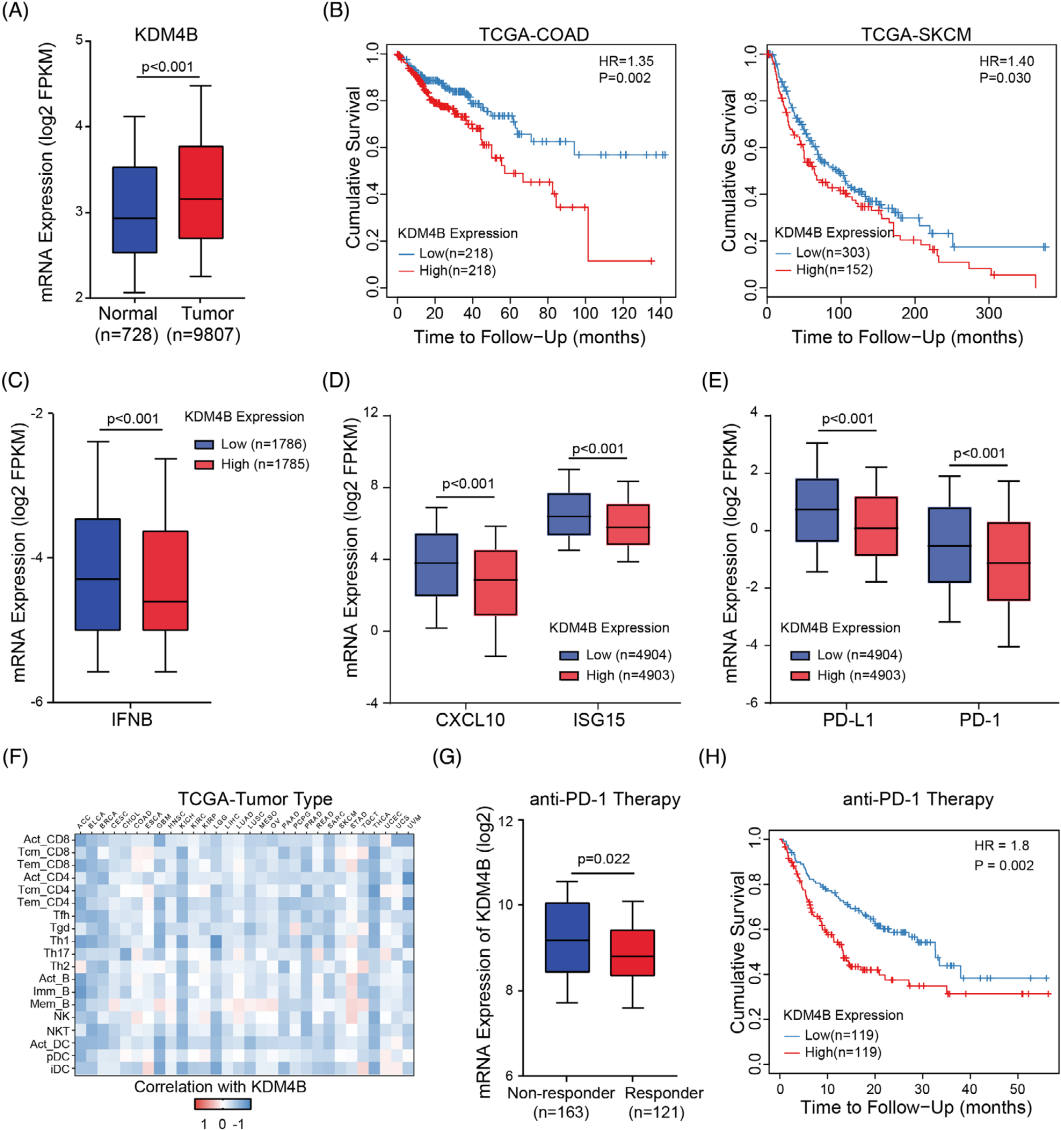
KDM4B expression is negatively correlated with type I interferon (IFN) signature and clinical outcomes. (A) The KDM4B gene expression was compared between tumour and normal tissues in The Cancer Genome Atlas (TCGA) pan‐cancer database. Normalized values were obtained from UCSC Xena database. (B) The relationship between KDM4B gene expression and overall survival prognosis of colorectal adenocarcinoma (COAD, left) and skin cutaneous melanoma (SKCM, right) in TCGA database. (C) The IFNB gene expression was compared between KDM4B‐low and KDM4B‐high tumour tissues in TCGA pan‐cancer database. Normalised values were obtained from UCSC Xena database. (D) The gene expression of CXCL10 and ISG15 was compared between KDM4B‐low and KDM4B‐high tumour tissues in TCGA pan‐cancer database. Normalised values were obtained from UCSC Xena database. (E) The gene expression of programmed death‐ligand 1 (PD‐L1) and PD‐1 was compared between KDM4B‐low and KDM4B‐high tumour tissues in TCGA pan‐cancer database. Normalised values were obtained from UCSC Xena database. (F) Heatmap shows the correlation between KDM4B gene expression and abundance of tumour‐infiltrating lymphocytes (TILs) in various tumour types in TCGA database. Data were obtained from the TISIDB database. (G) The KDM4B gene expression was compared between responder and non‐responder samples to anti‐PD‐1 therapy in pan‐cancer. Data were obtained from the ROC Plotter database and then log2‐transformed. (H) The relationship between KDM4B gene expression and overall survival prognosis of samples received anti‐PD‐1 therapy in pan‐cancer database. Data were obtained from Kaplan–Meier Plotter database. *p*‐Value in (A), (C–E) and (G) was calculated by unpaired Student's *t*‐test or log‐rank test in (B) and (H).

To determine the clinical significance of KDM4 family, we analysed their expression in human cancers. In TCGA pan‐cancer database, tumour tissues generally exhibited higher expression levels of KDM4A, KDM4B and KDM4D compared to normal tissues (Figures 7A and S12A). In contrast, KDM4C exhibited the opposite trend, with lower expression in tumour tissues (Figure S12A). Furthermore, survival analysis specifically highlighted that KDM4B expression consistently showed a negative correlation with overall survival in both colorectal adenocarcinoma (COAD) and skin cutaneous melanoma (SKCM) (Figures 7B and S12B). Given such unexpected and remarkable potency of KDM4B inhibition observed in both clinical settings and our experimental systems, we proceeded to shift our focus towards investigating the clinical relevance of KDM4B in human cancers. In COAD and SKCM Gene Expression Omnibus (GEO) cohorts, tumour tissues exhibited higher expression of KDM4B (Figure S12C). Additionally, we also evaluated KDM4B expression using colon cancer tissue microarrays, wherein we observed higher levels of KDM4B protein in colon tumour tissues compared to paired peri‐tumour specimens (Figure S12D). Classification of specimens into low and high KDM4B expression categories according to IHC scores revealed significantly shorter survival in patients with high KDM4B expression (Figure S12E), suggesting that KDM4B expression may serve as a prognostic marker.

To investigate the impact of KDM4B on the type I IFN signature in human cancers, we evaluated the levels of IFN‐β and downstream proteins. Notably, in the TCGA pan‐cancer database, samples exhibiting low KDM4B expression displayed elevated levels of IFN‐β, CXCL10, ISG15, PD‐L1 and PD‐1, in contrast to those with high KDM4B expression (Figure 7C–E). Moreover, a negative correlation was noticed between KDM4B expression and the expression of CXCL10 and ISG15 (Figure S12F). We next assessed the correlation between KDM4B expression and the abundance of tumour‐infiltrating immunocytes in 30 human cancers. Our analysis revealed a negative association between KDM4B expression and most immune cell infiltration, especially lymphocytes and dendritic cells in almost all cancers (Figure 7F), underscoring KDM4B's role in fostering an immunosuppressive TME. Furthermore, using the immunotherapy datasets, we observed lower expression of KDM4B in anti‐PD‐1 responders (Figure 7G). Remarkably, low expression of KDM4B was linked to improved overall survival in patients who received anti‐PD‐1 immunotherapy (Figure 7H), indicating a potent potential of KDM4B to differentiate between responders and non‐responders to anti‐PD‐1 immunotherapy. In general, our results demonstrate that KDM4B is commonly upregulated in cancer cells and highly correlated with the poor type I IFN signature, immunosuppressive TME, worse prognosis and inadequate response to immunotherapy.

## 4 DISCUSSION

Despite the emergence of immunotherapy as a successful clinical strategy for cancer treatment, a significant number of patients still remain unresponsive. Inducing a proper innate immune response may extend the benefits of T‐cell‐targeted immunotherapy to non‐responders. Herein, we identified that epigenetic inhibitor JIB‐04 significantly enhanced the tumour‐intrinsic innate immune response with remarkable antitumour effects. Specifically, by targeting KDM4 family, JIB‐04 induced H3K9 hypermethylation, which impeded DNA repair, caused DNA damage, promoted genomic and mitochondrial DNA leakage, and activated innate sensing via cytosolic DNA/cGAS/STING pathway. Importantly, JIB‐04‐induced DNA damage triggered an ICD and led to release of DAMPs, thereby activating innate and adaptive immunity. However, KDM4 inhibition in tumour cells also induced adaptive resistance by increasing the expression of PD‐L1. Combining anti‐PD‐L1 therapy effectively overcame this resistance and enhanced the antitumour effects of KDM4 inhibition.

JIB‐04 has previously been shown to exert cancer‐specific cytotoxicity.^21^ However, its role in immune regulation has not been reported. Our study has described the antitumour mechanism of JIB‐04 from the perspective of innate immunity. Interestingly, unlike KDM4, inhibiting the other two targets of JIB04, KDM5A and KDM6B, did not elicit any enhancing effects on type I IFN production, which was consistent with previous studies.^31^, ^32^ In our study, we utilised another KDM4 inhibitor, TACH101, to further validate that inhibiting KDM4 effectively activates type I IFNs and ICD. It is worth mentioning that TACH101 is presently undergoing phase I clinical trials for treating metastatic colorectal cancers with high microsatellite instability. It exhibited greater efficacy in enhancing type I IFN expression in human tumour cells compared to JIB‐04 (Figure S4D), highlighting its potential for clinical use and effectiveness in cancer treatment.

A significant limitation of conventional chemotherapy agents is their non‐tumour specificity, potentially causing unintended side effects due to their indiscriminate action on both cancerous and healthy cells.^33^ Previous studies have demonstrated that JIB‐04 induced cancer‐specific cell death.^21^, ^34^ This observation was consistent with clinical findings that the KDM4 family is often overexpressed in tumour tissue, highlighting the strong tumour specificity of JIB‐04. Furthermore, our in vivo results revealed no impairment in body weight, immune cell infiltration or CD8^+^ T‐cell activity following intratumoural administration of JIB‐04. In contrast, JIB‐04 significantly promoted immune cell infiltration and activation, thereby excluding negative impacts on the immune system and ensuring its safety in vivo.

Although type I IFN signatures are commonly induced by extracellular signals, their amplitudes, duration and gene expression patterns can be modulated by epigenetic factors.^35^ As a form of epigenetic modifications, histone demethylation has emerged as a pivotal regulator of type I IFNs and ISG expression. Depending on the particular genomic context and the level of methylation, histone demethylation may serve to either enhance or repress gene transcription.^36^ For example, KDM5B can suppress IFN‐β transcription by demethylating histone H3K4me3,^32^ whereas KDM6B functions as coactivator to increase IFN‐β transcription by demethylating histone H3K27me3,^37^ reflecting the specific actions of individual KDMs. In our study, our results demonstrate that KDM4 regulates the type I IFN expression via a distinct mechanism. By inducing H3K9 hypermethylation across the genome, KDM4 inhibition caused dsDNA stress and activated innate sensing to promote the transcription of type I IFNs. Indeed, H3K9me3 is critically involved in the response to DNA damage and the subsequent repair processes.^38^ When DNA damage occurs, H3K9 undergoes rapid methylation at the damaged site. This localised methylation signal facilitates the recruitment of DNA repair machinery, including tat‐interactive protein 60 and ataxia‐telangiectasia mutated protein. However, lack of H3K9‐specific demethylases results in widespread H3K9 methylation throughout the genome, which can obscure the local H3K9 methylation spike that normally occurs after DNA damage.^27^ This global hypermethylation can impair the recruitment of DNA repair factor, which can explain our observed suppression of DNA repair and promotion of DNA damage upon KDM4 inhibition.

Diminishing tumour burden, intensifying immunogenic reactions and invigorating innate immune responses are acknowledged as potent strategies to augment the efficacy of immunotherapy. Unlike traditional STING agonists that primarily stimulate innate immunity, JIB‐04 distinguishes itself through its dual mechanism of action. It not only provokes the STING pathway but also induces ICD, thereby simultaneously activating three critical pathways. This synergistic approach underscores the potential of JIB‐04 as a potent adjunct in immunotherapeutic regimens, orchestrating a comprehensive and multifaceted attack on tumour cells.

## 5 CONCLUSIONS

The ideal approach for cancer therapy is to activate the systematic immunity response while simultaneously killing cancer cells. Our study found that in addition to its direct cancer cell‐killing effects, KDM4 inhibition effectively triggers both innate and adaptive immunity. This stimulation occurs through mechanisms involving type I IFN and ICD, leading to outstanding antitumour effects. Remarkably, KDM4B exhibited the highest potential among all KDM4 family members in inducing type I IFN signalling and antitumour effects. However, KDM4 inhibition also induced adaptive immune resistance by increasing the expression of PD‐L1, which can be countered by combining it with anti‐PD‐L1 therapy. Our clinical analysis further revealed that KDM4B inhibition can significantly promote immune‐inflamed phenotypes and enhance the benefits of anti‐PD‐1 therapy. In summary, our findings demonstrate that KDM4 inhibition holds significant potential in promoting tumour‐cell‐intrinsic immune responses and enhancing the efficacy of immunotherapy.

## AUTHOR CONTRIBUTIONS

Xiaoguang Li and Hui Wang conceived the project. Mayu Sun, Xiaoyu Han and Jinyang Li designed experiments. Xiaoyu Han, Jinyang Li, Mayu Sun and Jingquan Li performed in vitro cell experiments. Xiaoyu Han, Jinyang Li and Mayu Sun performed in vivo mouse experiments. Xiaoyu Han analysed RNA‐seq and public TCGA data. Xiaoyu Han, Jinyang Li, Mayu Sun, Jiali Zheng and Xiaoguang Li wrote the manuscript. Xiaoguang Li and Hui Wang revised the manuscript. Xiaoguang Li and Hui Wang supervised the study.

## CONFLICT OF INTEREST STATEMENT

The authors declare they have no conflicts of interest.

## ETHICS STATEMENT

Human tissue microarray of colon cancer was obtained from Shanghai Outdo Biotech Company (Shanghai, China) with the approval of the Institutional Review Board. All animal experiments were conducted in accordance with protocols approved by the Institutional Animal Care and Use Committee of Shanghai Jiao Tong University School of Medicine (approval number: A‐2020‐001).

## Supporting information

Supporting Information

## Data Availability

All data relevant to the study are included in the article or uploaded as Supporting Information.
