# Supplementary material for: Targeting KDM4 family epigenetically triggers antitumour immunity via enhancing tumour‐intrinsic innate sensing and immunogenicity
Source: Clin Transl Med. 2024 Feb 23;14(2):e1598. doi: 10.1002/ctm2.1598 (PMC10884983; doi:10.1002/ctm2.1598)
Supplement: Supplementary file 1 — Supporting Information [file CTM2-14-e1598-s001.docx]

**Supplementary Information for**

**Targeting KDM4 family epigenetically triggers antitumor immunity via enhancing tumor-intrinsic innate sensing and immunogenicity**

Mayu Sun^1,#^, Xiaoyu Han^1,#^, Jinyang Li^1,#^, Jiali Zheng^2^, Jingquan Li^1^, Hui Wang^1,*^ and Xiaoguang Li^1,*^

*^1^State Key Laboratory of Systems Medicine for Cancer, Center for Single-Cell Omics, School of Public Health, Shanghai Jiao Tong University School of Medicine, Shanghai, China;*

*^2^ Department of Epidemiology and Biostatistics, School of Public Health, Shanghai Jiao Tong University School of Medicine, Shanghai, China*

^#^These authors contributed equally.

**Correspondence:** Dr. Hui Wang or Dr. Xiaoguang Li, State Key Laboratory of Systems Medicine for Cancer, Center for Single-Cell Omics, School of Public Health, Shanghai Jiao Tong University School of Medicine, Shanghai 200025, China; Email: huiwang@shsmu.edu.cn (H. Wang) or lixg@shsmu.edu.cn (X. Li).


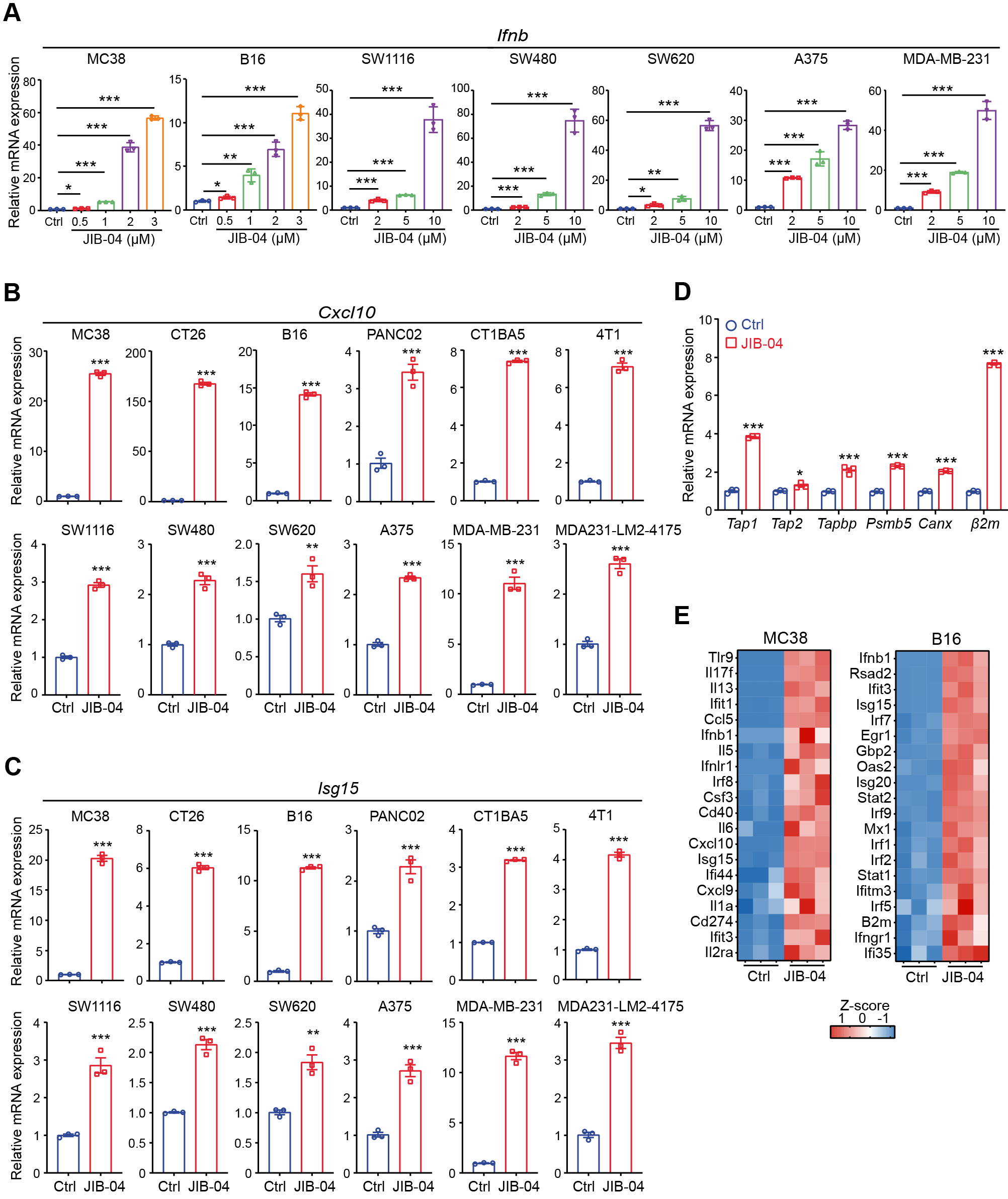


**Supplementary Fig 1. JIB-04 promotes the expression of IFN-β and its downstream genes in both human and mouse cells**. (**A**) Indicated mouse and human tumor cells were treated with indicated dose of JIB-04 for 24 hours. Cells were collected for IFN-β mRNA detection by RT-qPCR. (**B, C**) Indicated mouse and human tumor cells were treated with JIB-04 (2 µM) for 24 hours, and cells were collected for Cxcl10 (**B**) and Isg15 (**C**) mRNA detection by RT-qPCR. (**D**) MC38 cells were treated with JIB-04 (2 µM) for 24 hours. Cells were collected for indicated mRNA detection by RT-qPCR. (**E**) Heatmaps displaying the changes in type I IFN-related genes following treatment with JIB-04 in MC38 (left) and B16 (right) cells based on RNA-Seq data. Data are shown as mean ± SEM (n=3). *P* value was calculated by unpaired Student's t-test. (*p < 0.05, ***p < 0.01, ***p < 0.001).


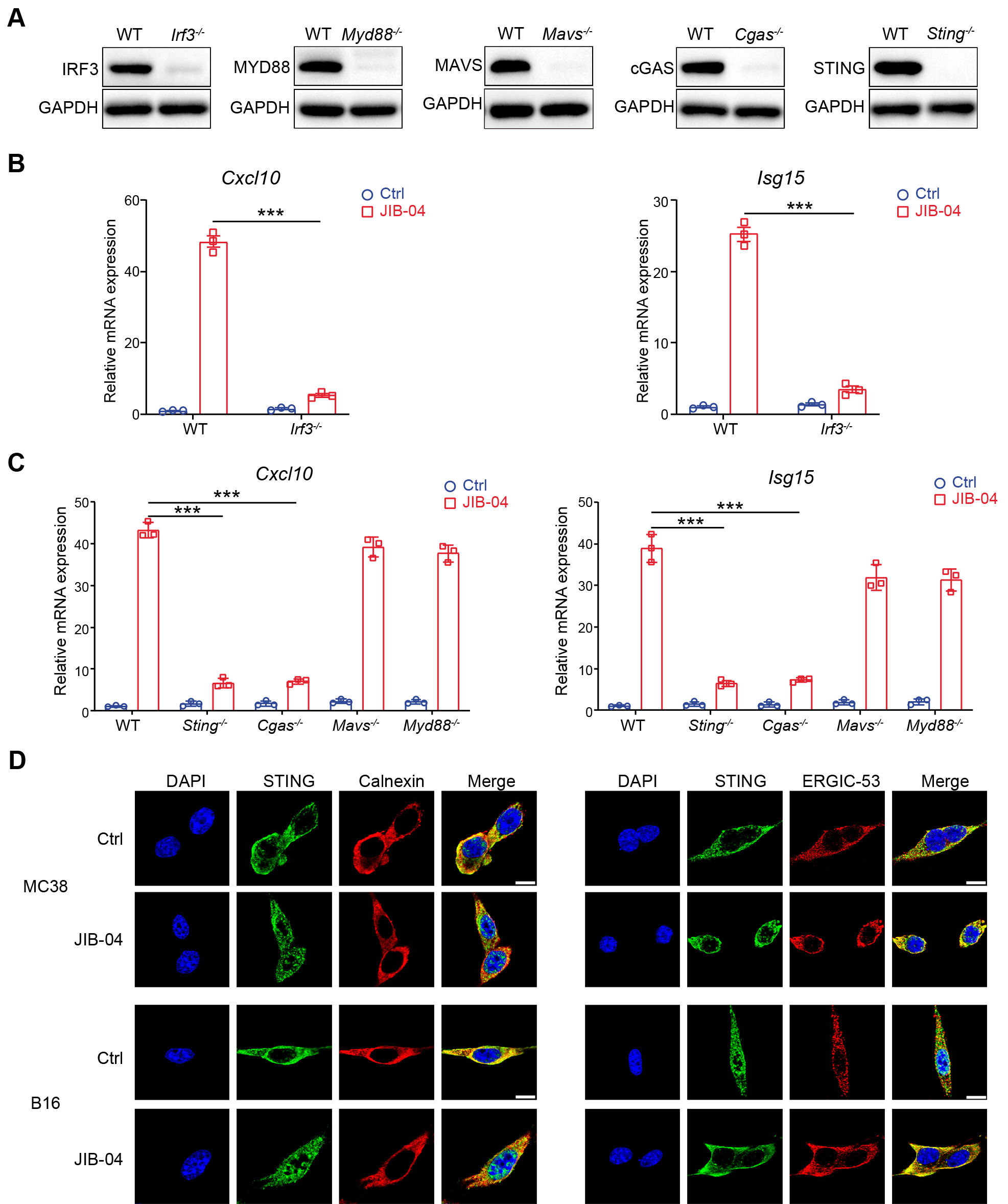


**Supplementary Fig 2. JIB-04 activates type I IFN signaling via cGAS/STING pathway**. (**A**) The knockout efficiency of target genes in MC38 cells was determined by western blot. (**B**) WT and Irf3-deficient MC38 cells were treated with 2 µM JIB-04 for 24 hours, and the mRNA level of IFN-β was determined by RT-qPCR. (**C**) MC38 cells with indicated gene knockout were treated with 2 µM JIB-04 for 24 hours, and the mRNA levels of Cxcl10 and Isg15 were determined by RT-qPCR. (**D**) Representative confocal images showing STING (green), ER marker-Calnexin/ERGIC marker-ERGIC-53 (red) and nuclei (blue) in MC38 or B16 cells after JIB-04 treatment (2 µM, 24 hours). Scale bar, 5 µm. Data are shown as mean ± SEM (n=3). *P* value was calculated by unpaired Student's t-test. (***p < 0.001).

**
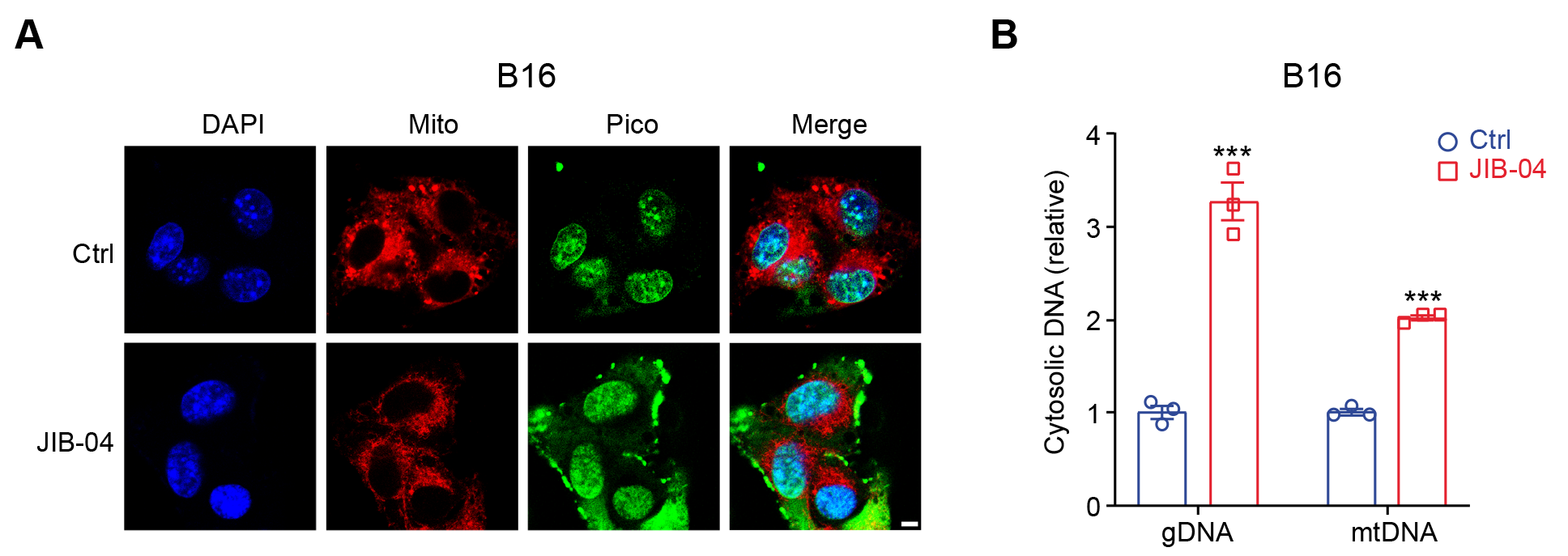
Supplementary Fig 3. JIB-04 promotes the accumulation of cytosolic DNA in B16 cells**. (**A**) Representative confocal images showing cytosolic dsDNA (green), mitochondria (red) and nuclei (blue) in B16 cells after JIB-04 treatment (2 µM, 24 hours). Scale bar, 5 µm. (**B**) B16 cells were treated with 2 µM JIB-04 for 24 hours, and the levels of gDNA and mtDNA were determined by RT-qPCR. Data are shown as mean ± SEM (n=3). *P* value was calculated by unpaired Student's t-test. (***p < 0.001).


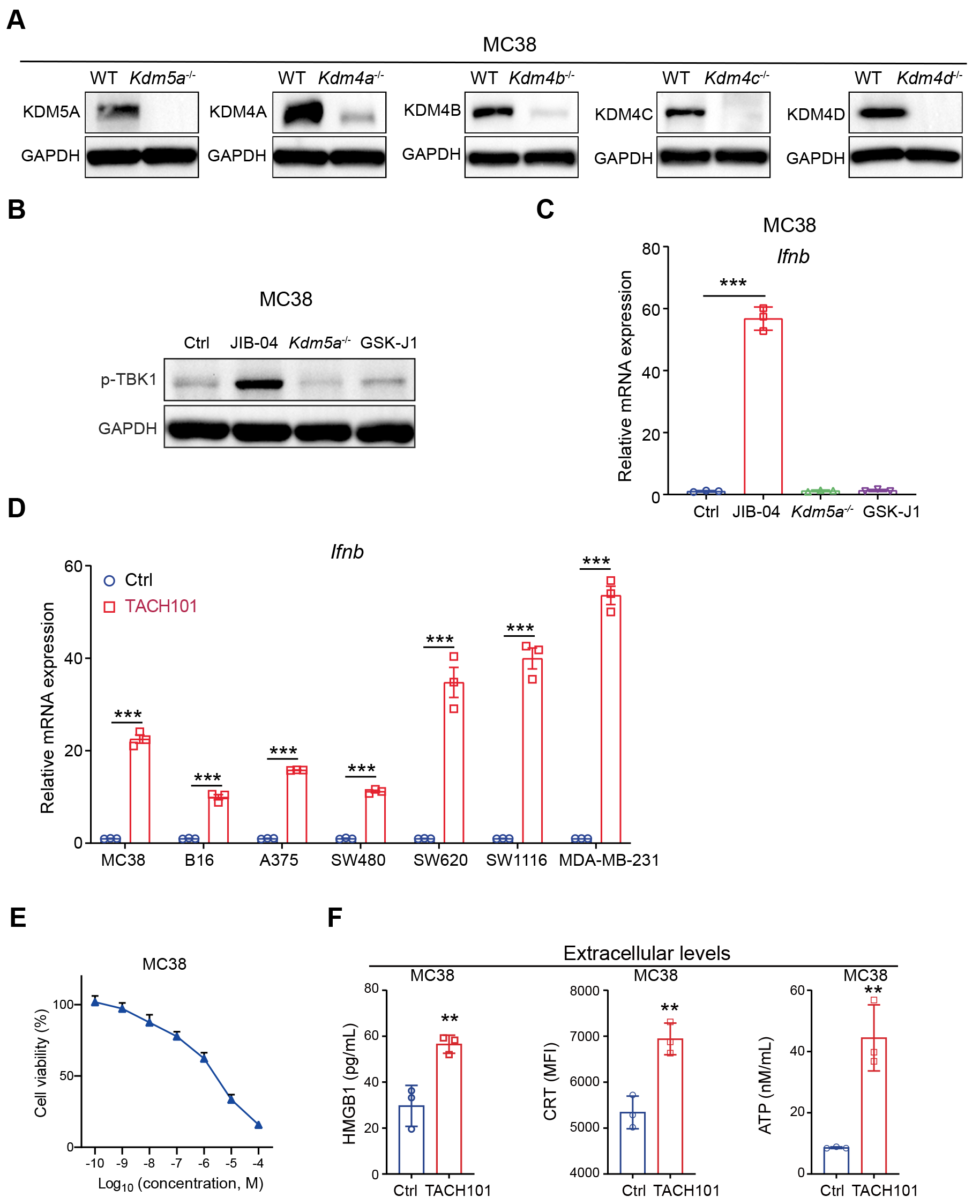


**Supplementary Fig 4. Unlike KDM4, inhibition of KDM5A and KDM6B does not affect TBK1 activation and IFN-β production.** (**A**) The knockout efficiency of target genes in MC38 cells was determined by western blot. (**B**) The protein level of p-TBK1 in JIB-04-treated, Kdm5a-deficient, and GSK-J1 (10 µM)-treated MC38 cells was determined by western blot. (**C**) The mRNA level of IFN-β in JIB-04-treated, Kdm5a-deficient, and GSK-J1-treated MC38 cells was determined by RT-qPCR. (**D**) Indicated mouse and human tumor cells were treated with TACH101 (2 µM) for 24 hours. Cells were collected for IFN-β mRNA detection by RT-qPCR. (**E**) MC38 cells were treated with TACH101 at various concentrations for 24 hours, and cell viability was determined by CCK-8 assay. (**F**) The extracellular levels of HMGB1 (left) and ATP (right) were detected by ELISA, and the extracellular expression of CRT (middle) was detected by flow cytometry. Data are shown as mean ± SEM (n=3). *P* value was calculated by unpaired Student's t-test. (**p < 0.01, ***p < 0.001).


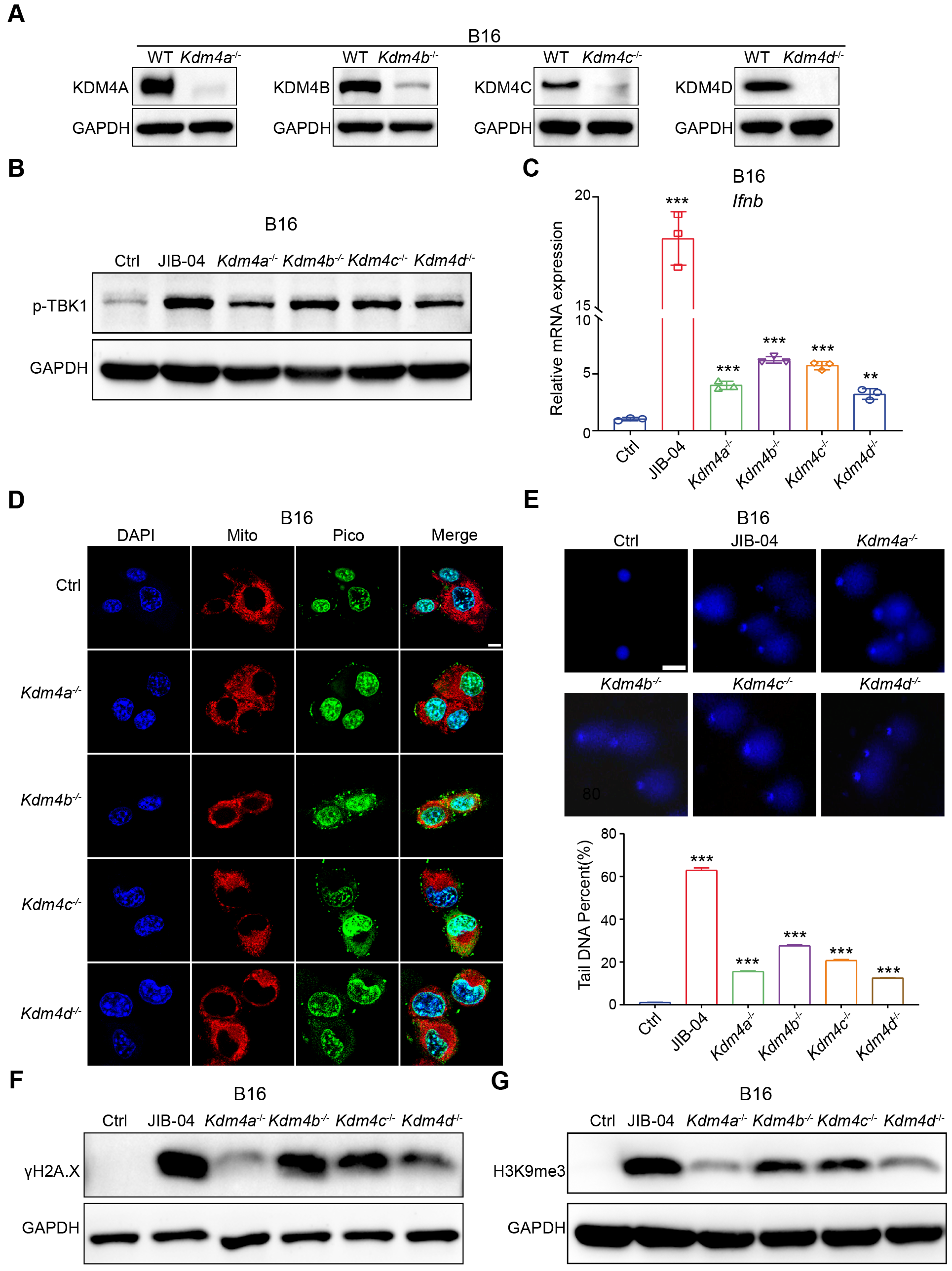


**Supplementary Fig 5. Genetic deletion of KDM4 family genes promotes DNA damage and activates tumor-intrinsic DNA sensing in B16 cells**. (**A**) The knockout efficiency of target genes in B16 cells was determined by western blot. (**B, C**) Indicated genes were knocked out in MC38 cells, and the protein level of p-TBK1 (**B**) was determined by western blot; the mRNA level of IFN-β (**C**) was determined by RT-qPCR (n=3). (**D**) Representative confocal images showing cytosolic dsDNA (green), mitochondria (red) and nuclei (blue) in B16 cells with indicated gene knockout. Scale bar, 10 µm. (**E**) Representative images (top) and quantification (bottom) of DNA comet assays in B16 cells with indicated gene knockout. More than 100 cells were analyzed in every group. Scale bar, 50 µm. (**F**, **G**) The protein levels of γH2A.X (**F**) and H3K9me3 (**G**) in MC38 cells with indicated gene knockout were determined by western blot. Data are shown as mean ± SEM. *P* value was calculated by unpaired Student's t-test. (**p < 0.01, ***p < 0.001).


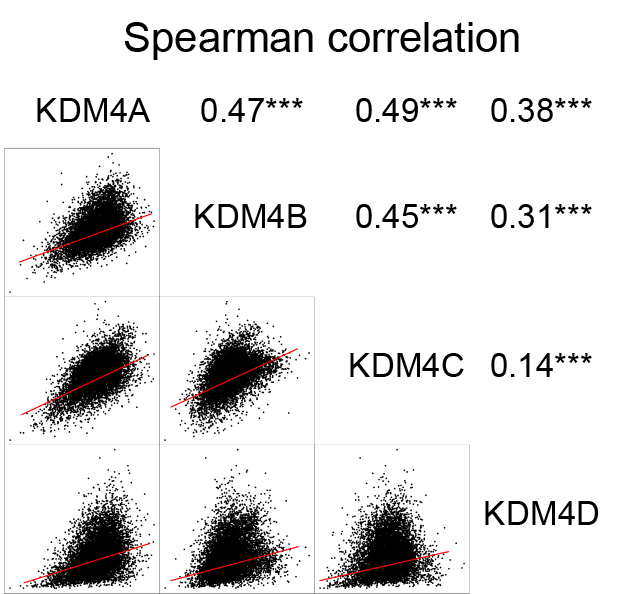


**Supplementary Fig 6. The Correlation among the expression of KDM4A, KDM4B, KDM4C, and KDM4D in TCGA pan-cancer database.** Correlations and significance were calculated using Spearman's rank correlation analysis (***p < 0.001).


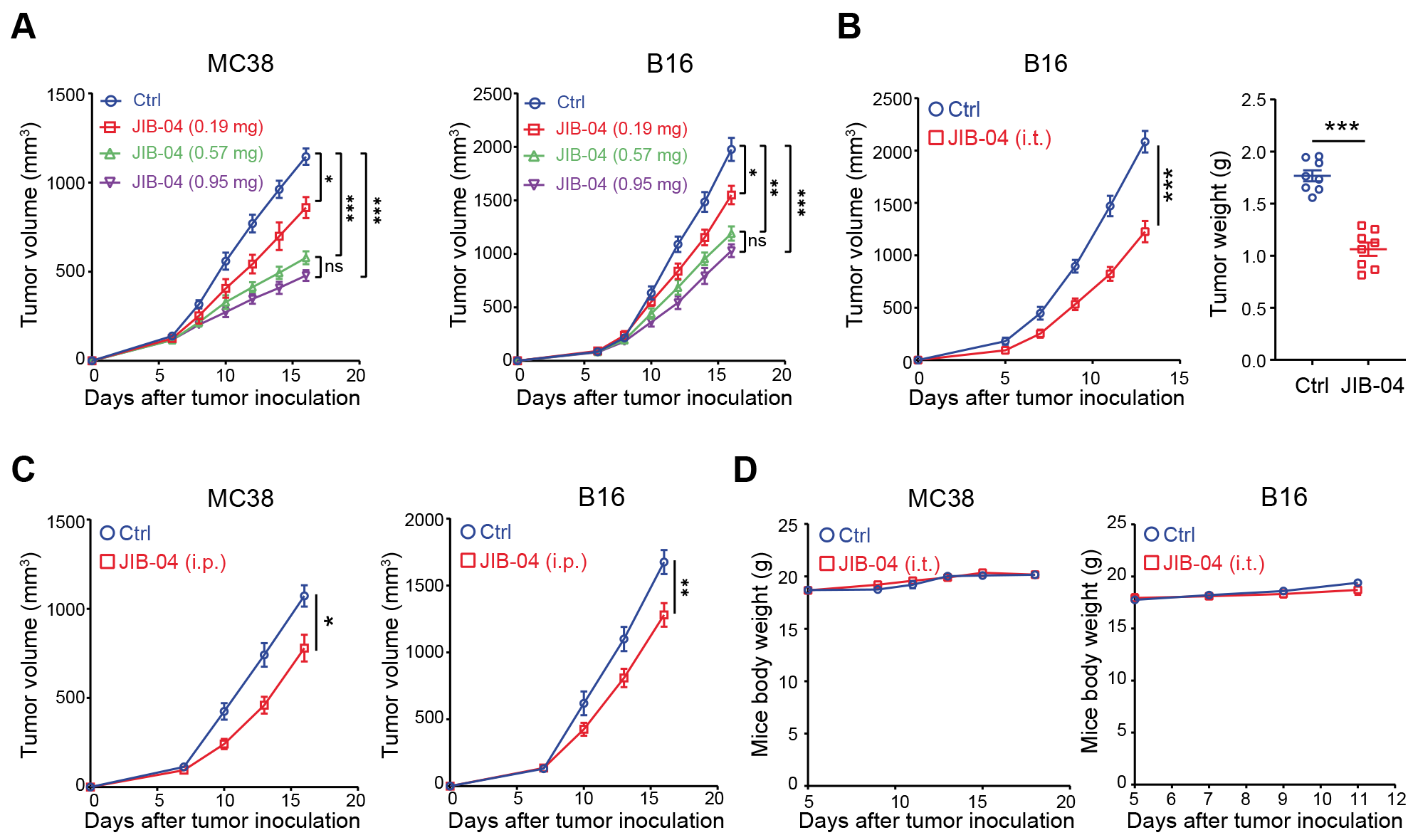


**Supplementary Fig 7. Local and systemic administration of JIB-04 effectively control tumor growth without obvious effects on body weight of mice**. (**A**) C57BL/6 mice (n=8 per group) were inoculated with MC38 (left) or B16 (right) cells and subjected to intratumoral treatment with JIB-04 at various daily doses (0.19, 0.57, and 0.95 mg per injection). Tumor growth were measured. (**B**) C57BL/6 mice (n=8 per group) were inoculated with B16 cells and subjected to intratumoral treatment with JIB-04. Tumor growth (left) and weights (right) were measured. (**C**) C57BL/6 mice (n=8 per group) were inoculated with MC38 (left) or B16 (right) cells and subjected to intraperitoneal treatment with JIB-04 (30 mg/kg). Tumor growth were measured. (**D**) The body weight changes of MC38 (left) and B16 (right) tumor-bearing mice during intratumoral treatment with JIB-04. Data are shown as mean ± SEM. *P* value was calculated by unpaired Student's t-test in **B** or two-way ANOVA in **A**-**C**. (*p < 0.05, **p < 0.01, ***p < 0.001).


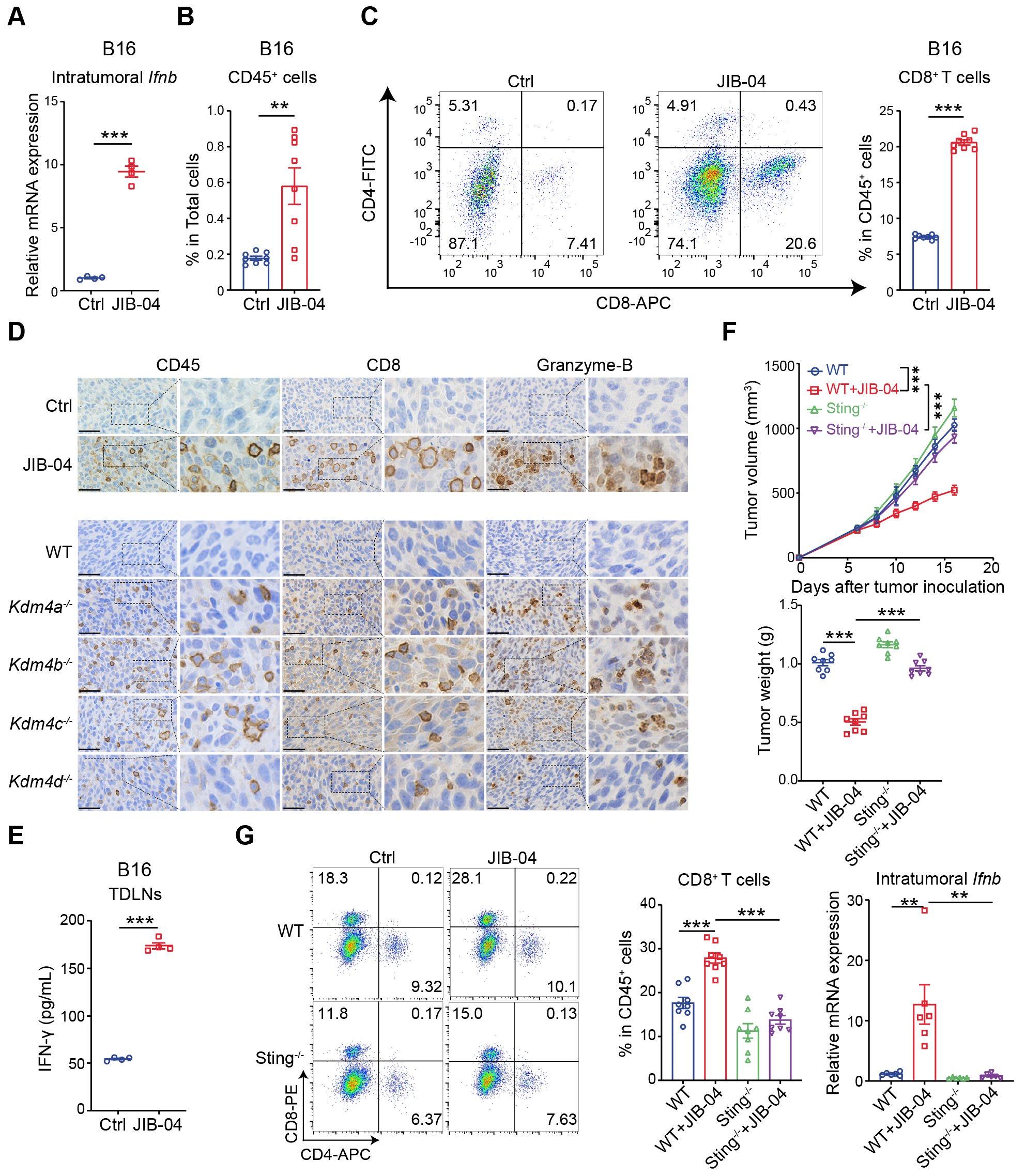


**Supplementary Fig 8. Pharmacological or genetic inhibition of KDM4 promotes antitumor immunity**. (**A**) The mRNA expression of IFN-β in B16 tumor tissues was determined by RT-qPCR. (**B**) The proportion of infiltrating CD45^+^ cells in the TME of B16 tumor-bearing mice was detected by flow cytometry. (**C**) Representative flow cytometry plots (left) and quantification (right) showing the infiltration proportion of CD8^+^ T cells in the TME of B16 tumor-bearing mice. (**D**) Representative immunohistochemistry images showing the proportion of tumor-infiltrating cells and CD8^+^ T cells as well as the expression of Granzyme B in MC38 tumors after indicated treatments. scale bar=40µm. (**E**) The tumor-draining lymph nodes (TLNs) of B16 tumor-bearing mice were dissected, and then dissolved into suspensions. After re-stimulated with dead B16 cells for 48 hours, the supernatant was collected for IFN-γ detection by ELISA. (**F**) WT or Sting-deficient MC38 tumor-bearing mice (n=8 per group) were treated with JIB-04. Tumor growth and weights were measured. (**G**) Representative flow cytometry plots (left) and quantification (middle) showing the infiltration proportion of CD8^+^ T cells in the TME of MC38 tumor-bearing mice. The mRNA expression of IFN-β (right) in indicated tumor tissues was determined by RT-qPCR. Data are shown as mean ± SEM. *P* value was calculated by unpaired Student's t-test in **A**-**C, E-G** or two-way ANOVA in **F**. (**p < 0.01, ***p < 0.001).


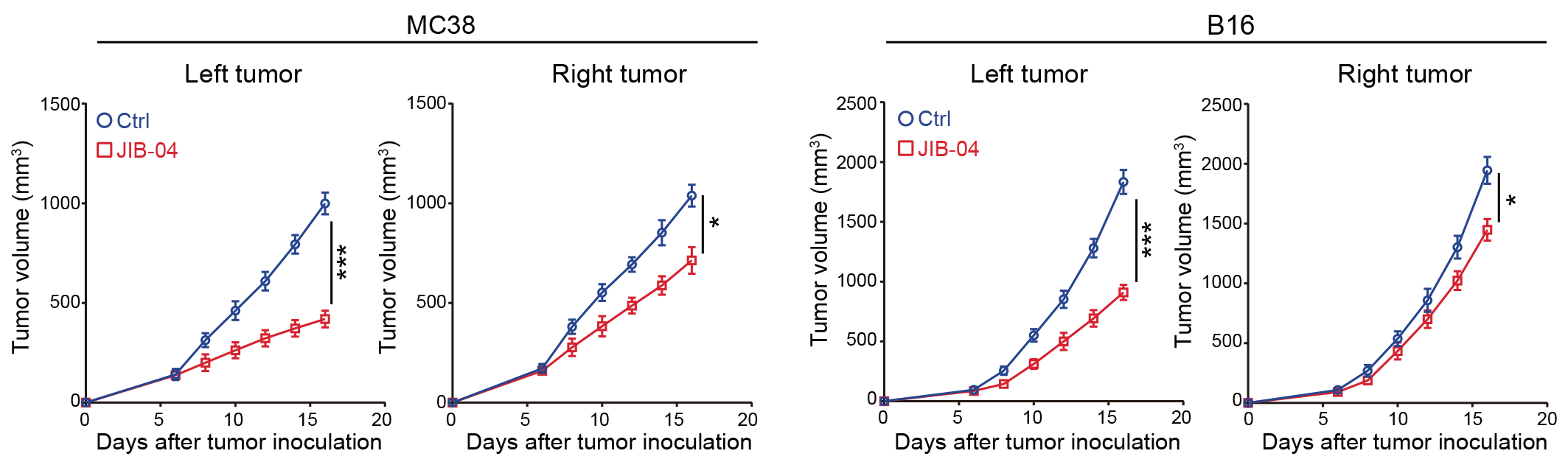


**Supplementary Fig 9. JIB-04 triggers systemic antitumor responses.** C57BL/6 mice (n=8 per group) were inoculated with MC38 or B16 cells on both the left and right flanks. The left tumor received intratumoral injection of JIB-04, while the right tumor remained untreated. The growth of both the left and right tumors was monitored. Data are shown as mean ± SEM. *P* value was calculated by two-way ANOVA. (*p < 0.05, ***p < 0.001).


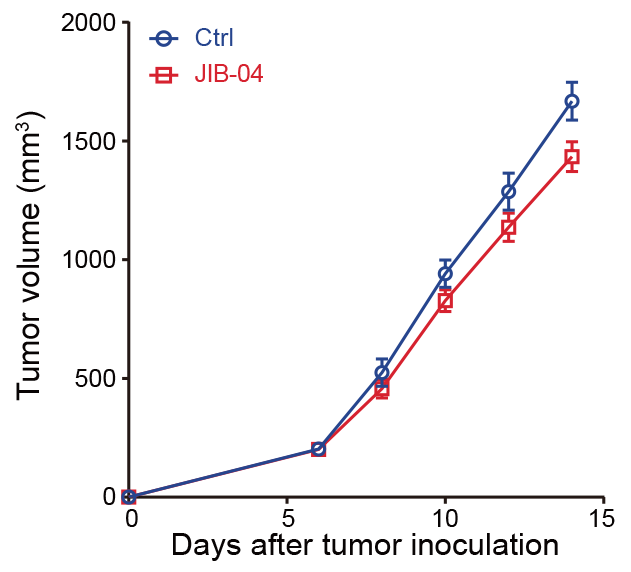


**Supplementary Fig 10. Antitumor effects of JIB-04 depend on T cells.** BALB/c nude mice (n=8 per group) were inoculated with MC38 cells and subjected to intratumoral treatment with JIB-04. Tumor growth was measured.


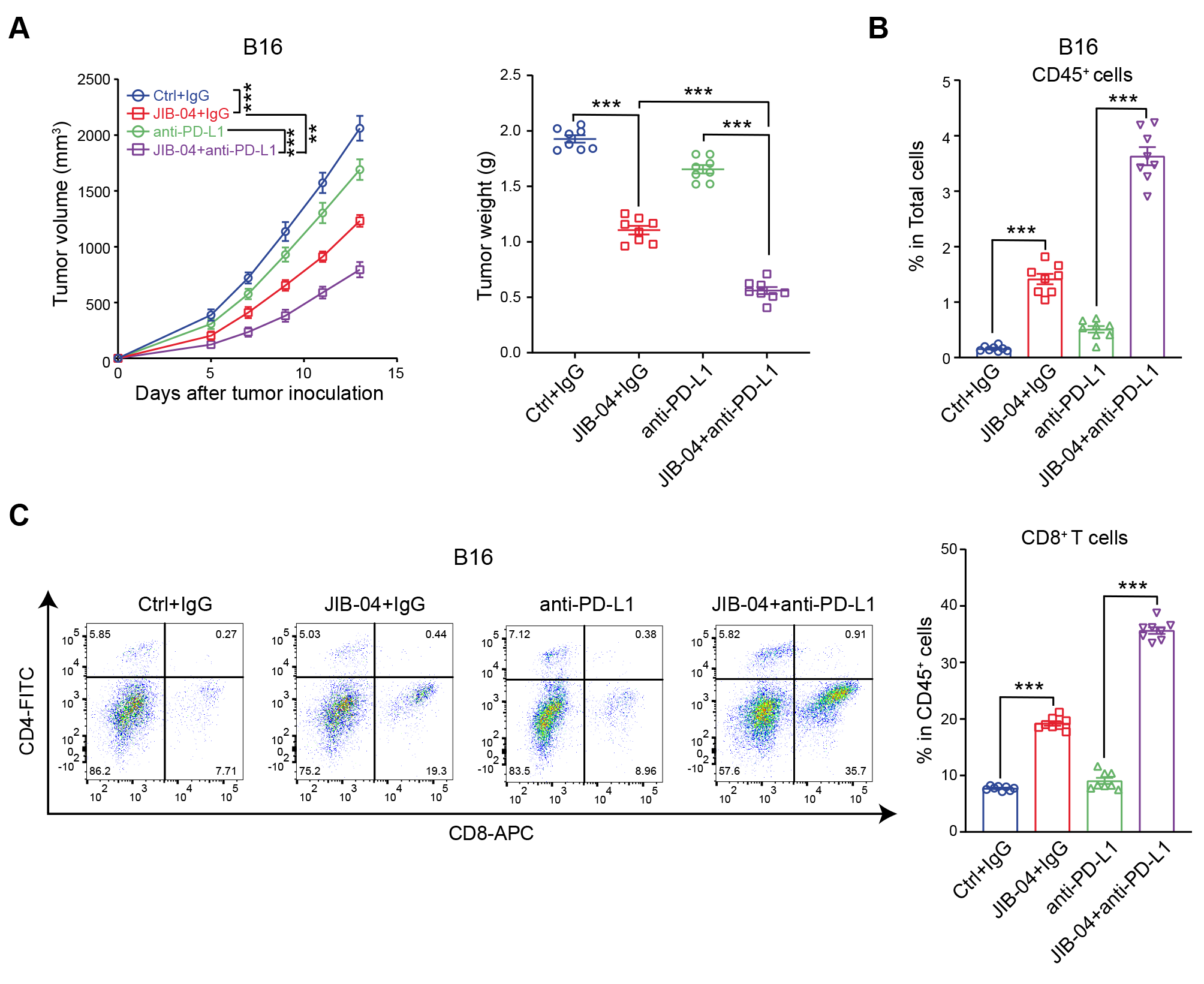


**Supplementary Fig 11. JIB-04** **re-sensitizes B16 tumors to PD-L1 blockade**. (**A**) C57BL/6 mice (n=8/group) were inoculated with 5×10^5^ B16 cells and subjected to JIB-04 treatment and/or anti-PD-L1 antibodies. Tumor growth (left) and weights (right) were measured. (**B**) The proportion of infiltrating CD45^+^ cells in the TME of B16 tumor-bearing mice was detected by flow cytometry. (**C**) Representative flow cytometry plots (left) and quantification (right) showing the infiltration proportion of CD8^+^ T cell in the TME of B16 tumor-bearing mice. Data are shown as mean ± SEM. *P* value was calculated by unpaired Student's t-test in **A-C** or two-way ANOVA in **A**. (**p < 0.01, ***p < 0.001).


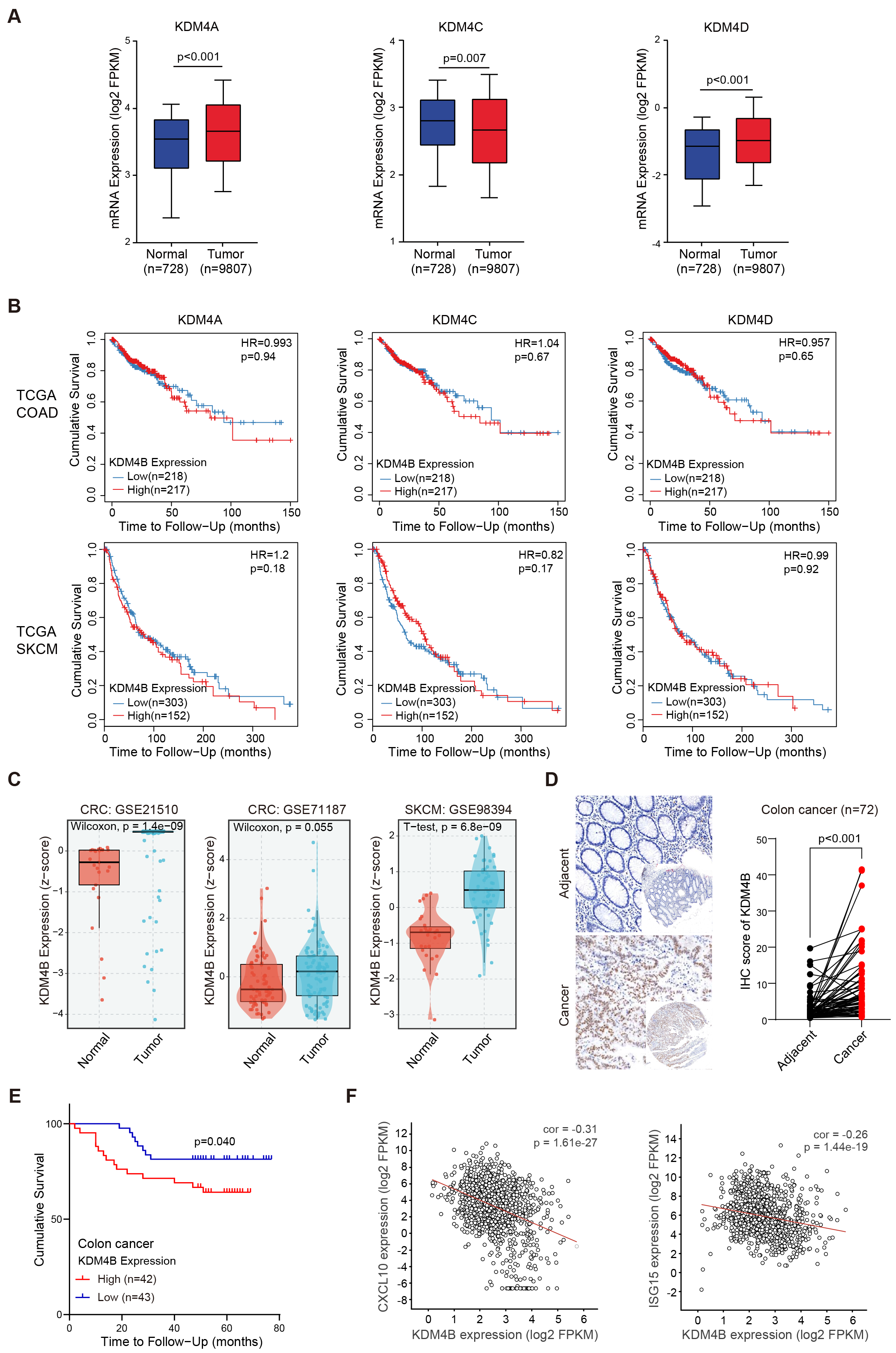


**Supplementary Fig 12. KDM4B is overexpressed in cancer cells and negatively correlated with clinical outcomes and type I IFN signature**. (**A**) The gene expression of KDM4A, KDM4C and KDM4D was compared between tumor and normal tissues in TCGA pan-cancer database. Normalized values were obtained from UCSC Xena database. (**B**) The relationship between KDM4A/C/D gene expression and overall survival prognosis of colorectal adenocarcinoma (COAD, top) and skin cutaneous melanoma (SKCM, bottom) in TCGA database. (**C**) The gene expression of KDM4B was compared between tumor and normal tissues in colorectal cancer (CRC) and SKCM GEO database. Statistical significance was calculated by Student's t-test and Wilcoxon. All data of GSE21510, 71187, and 98394 were reanalyzed using the BEST database (<https://rookieutopia.com/>). (**D**) Representative images from immunohistochemical staining of KDM4B in colon cancer and matched adjacent tissue. KDM4B protein levels in cancer tissues are compared with matched adjacent normal tissues in colon cancer by using a Wilcoxon matched pairs test. (**E**) Kaplan-Meier plot of overall survival of patients with colon cancer stratified by the KDM4B expression level. A log-rank test was used for statistical analysis. (**F**) The correlation between the expression of KDM4B and the expression of CXCL10 (left) as well as ISG15 (right) in TCGA pan-cancer database. Correlations and significance were calculated using Spearman's rank correlation analysis.

**Supplementary table 1. sgRNA targeting sequences for CRISPR/Cas9-mediated gene knockout**

| **Target gene** | **sgRNA sequence** |
| --- | --- |
| *Ctrl* | GTGAGCCTATATTAATTGG |
| *Irf3* | ACGTCCGGCTTATCCTTCCC |
| *Sting* | CAGCAAAACATCGACCGTGC |
| *cGAS* | CGCAAAGGGGGGCTCGATCG |
| *Mavs* | GCCGTCGCGAGGATGTCTGG |
| *Myd88* | CGCATATAGTGATGAACCGC |
| *Kdm5a* | AACAAGACGAGTTAAGTCTC |
| *Kdm4a* | TAGATCATCAATATCGTCGT |
| *Kdm4b* | CGAATGCGGGACCATCATTG |
| *Kdm4c* | CGACTTGAAAGACTAGCCCA |
| *Kdm4d* | TGCTTTGGTCACCCGCCGGG |

**Supplementary table 2. Primers for qRT-PCR**

| **Species** | **Gene** | **Forward sequence** | **Reverse sequence** |
| --- | --- | --- | --- |
| Mouse | *Ifnβ* | ATGAGTGGTGGTTGCAGGC | TGACCTTTCAAATGCAGTAGATTCA |
|  | *Cxcl 10* | GGAGTGAAGCCACGCACAC | ATGGAGAGAGGCTCTCTGCTGT |
|  | *Isg 15* | GAGCTAGAGCCTGCAGCAAT | TCACGGACACCAGGAAATCG |
|  | *L32* | TTAAGCGAAACTGGCGGAAAC | TTGTTGCTCCCATAACCGATG |
|  | *Tert* | CTAGCTCATGTGTCAAGACCCTCTT | GCCAGCACGTTTCTCTCGTT |
|  | *Dloop1* | AATCTACCATCCTCCGTGAAACC | TCAGTTTAGCTACCCCCAAGTTTAA |
|  | *Tap1* | CTCTTGGTGTTCATGTTTTGGG | CGTGGACTTTGCTAGAGACTC |
|  | *Tap2* | TCGTGTAATTGACATCCTGGG | CTGGACATGGTGAAGAGGAAG |
|  | *Tapbp* | AGCAGCATGGAGTTCACTATG | AGGAGAAAAGCAGACAGGAAC |
|  | *Psmb5* | CGAATCGAAATGCTTCACGG | CAGAAGGTACGGGTTGATCTC |
|  | *Canx* | AAGCCCTTCCTGTTTGATACC | AGTATAGGGAGTCTTGTCGTGG |
|  | *β2m* | TGGTCTTTCTGGTGCTTGTC | GGGTGGAACTGTGTTACGTAG |
|  | *Ifnγ* | ATGAACGCTACACACTGCATC | CCATCCTTTTGCCAGTTCCTC |
|  | *Pd-l1* | GCTCCAAAGGACTTGTACGTG | TGATCTGAAGGGCAGCATTTC |
|  | *Actb* | AGAGGGAAATCGTGCGTGAC | CAATAGTGATGACCTGGCCGT |
| Human | *IFNβ* | AGTAGGCGACACTGTTCGTG | AGCCTCCCATTCAATTGCCA |
|  | *CXCL10* | CCACGTGTTGAGATCATTGCT | TGCATCGATTTTGCTCCCCT |
|  | *ISG15* | ACAGCCATGGGCTGGGAC | GGTTCGTCGCATTTGTCCAC |
|  | *β-ACTIN* | CTCGCCTTTGCCGATCC | TCTCCATGTCGTCCCAGTTG |

**Supplementary table 3. A collection of 176 epigenetics inhibitor compounds**

| Index | Compound | Target |
| --- | --- | --- |
| 1 | EPZ004777 | Histone Methyltransferase inhibitor |
| 2 | Arsenic oxide (3) | Others |
| 3 | Carvedilol | Potassium Channel inhibitor; VEGF; Vcam inhibitor; E-selectin inhibitor |
| 4 | Ellagic acid | Casein Kinase inhibitor; Carbonic anhydrase inhibitor; PKA inhibitor |
| 5 | Procainamide hydrochloride | ChE inhibitor; DNA Methyltransferase inhibitor; Sodium Channel inhibitor |
| 6 | Theophylline monohydrate | Adenosine Receptor antagonist; HDAC2 activator; PDE inhibitor |
| 7 | Fluorouracil (5-Fluoracil, 5-FU) | HIV Integrase; DNA/RNA Synthesis inhibitor |
| 8 | Doxorubicin hydrochloride | Topoisomerase inhibitor |
| 9 | Tranylcypromine (2-PCPA) hydrochloride | Histone Demethylase inhibitor; MAO inhibitor |
| 10 | Docetaxel | Bcl-2 inhibitor; Microtubule Assosiated inhibitor |
| 11 | Carboplatin | DNA Synthesis inhibitor |
| 12 | Theophylline | PDE inhibitor; HDAC activator; Adenosine receptor antagonist |
| 13 | Norfloxacin | Topoisomerase inhibitor; DNA gyrase inhibitor |
| 14 | Mylosar | DNA Methyltransferase inhibitor |
| 15 | Temozolomide | DNA Alkylating |
| 16 | Mizoribine | DNA/RNA Synthesis inhibitor |
| 17 | Lovastatin | HDAC inhibitor; HMG-CoA reductase inhibitor; Integrin |
| 18 | Vincristine sulfate | Microtubule Associated inhibitor; MRP inhibitor |
| 19 | Cytarabine | DNA Synthesis inhibitor |
| 20 | Olaparib (AZD2281, Ku-0059436) | PARP inhibitor |
| 21 | Oxfendazole | Microtubule Associated inhibitor; Glucose uptake inhibitor |
| 22 | Ofloxacin | Topoisomerase inhibitor |
| 23 | Fisetin | Sirtuin activator |
| 24 | Tamoxifen citrate | Estrogen/Progestogen Receptor agonist |
| 25 | Zebularine | DNA Methyltransferase inhibitor |
| 26 | Quercetin | Sirtuin inhibitor |
| 27 | Entacapone | Histone Methyltransferase inhibitor |
| 28 | Sodium Phenylbutyrate | HDAC inhibitor |
| 29 | Rapamycin (Sirolimus) | mTOR inhibitor; FGFR inhibitor |
| 30 | Resveratrol | HDAC activator; DNA Synthesis inhibitor; COX inhibitor |
| 31 | Cisplatin | Caspase activator; DNA/RNA Synthesis inhibitor |
| 32 | Tofacitinib (CP-690550) Citrate | JAK inhibitor |
| 33 | SGC0946 | Histone Methyltransferase inhibitor |
| 34 | Bortezomib (PS-341) | Protease inhibitor |
| 35 | Vorinostat (SAHA, MK0683) | HDAC inhibitor |
| 36 | Valerin | GABAR; HDAC inhibitor |
| 37 | Aminophylline | PDE inhibitor; Adenosine receptor antagonist; HDAC2 activator |
| 38 | Daptomycin | Antibiotic; DNA/RNA Synthesis inhibitor |
| 39 | BET bromodomain inhibitor | Epigenetic Reader Domain inhibitor |
| 40 | AMI-1 | Histone Methyltransferase inhibitor |
| 41 | UNC1215 | Epigenetic Reader Domain antagonist |
| 42 | Danusertib (PHA-739358) | Aurora Kinase inhibitor |
| 43 | EPZ6438 | Histone Methyltransferase inhibitor |
| 44 | BET bromodomain inhibitor | Epigenetic Reader Domain inhibitor |
| 45 | Panobinostat (LBH589) | HDAC inhibitor |
| 46 | GSK126 | Histone Methyltransferase inhibitor |
| 47 | CUDC-907 | HDAC inhibitor |
| 48 | GSK503 | Histone Methyltransferase inhibitor |
| 49 | UNC0379 | Histone Methyltransferase inhibitor |
| 50 | MLN8237(Alisertib) | Aurora Kinase inhibitor |
| 51 | GSK-LSD1 dihydrochloride | Histone Demethylase inhibitor |
| 52 | SP2509 | Histone Demethylase inhibitor |
| 53 | PFI-2 | Histone Methyltransferase inhibitor |
| 54 | ABT888 hydrochloride | PARP inhibitor |
| 55 | (+)-JQ-1 | Epigenetic Reader Domain inhibitor |
| 56 | WHI-P154 | JAK inhibitor; EGFR inhibitor |
| 57 | UNC669 | Epigenetic Reader Domain antagonist |
| 58 | PJ34 hydrochloride | PARP inhibitor |
| 59 | ENMD-2076 | Aurora Kinase inhibitor |
| 60 | PCI-24781 (Abexinostat) | HDAC inhibitor |
| 61 | Decernotinib(VX-509) | JAK inhibitor |
| 62 | EPZ011989 | Histone Methyltransferase inhibitor |
| 63 | TAK901 | Aurora Kinase inhibitor; JAK inhibitor; |
| 64 | Fasudil (HA-1077) hydrochloride | ROCK inhibitor |
| 65 | SMI-4a | Pim inhibitor |
| 66 | UNC1999 | Histone Methyltransferase inhibitor |
| 67 | Histone Deacetylase Inhibitor III | HDAC inhibitor |
| 68 | Fedratinib (SAR302503, TG101348) | JAK inhibitor; FLT3 inhibitor; RET inhibitor |
| 69 | (-)-Epigallocatechin Gallate | telomerase; DNA methyltransferase inhibitor |
| 70 | Daphnetin | EGFR inhibitor; PKC inhibitor |
| 71 | EPZ5676 | Histone Methyltransferase inhibitor |
| 72 | CUDC-101 | HDAC inhibitor |
| 73 | 2-Amino-6-mercaptopurine | DNA Methyltransferase inhibitor |
| 74 | Entinostat (MS-275) | HDAC inhibitor |
| 75 | Irinotecan | Topoisomerase inhibitor |
| 76 | Iniparib (BSI-201) | PARP inhibitor |
| 77 | Imatinib (STI571) | PDGFR inhibitor |
| 78 | GSK J4 hydrochloride | Histone Demethylase inhibitor |
| 79 | PJ34 | PARP inhibitor |
| 80 | Procarbazine hydrochloride | DNA Synthesis inhibitor |
| 81 | Paclitaxel | Bcl-2 inhibitor; Microtubule Assosiated inhibitor |
| 82 | Etoposide | Topoisomerase inhibitor; MRP inhibitor |
| 83 | Methotrexate | DHFR inhibitor |
| 84 | TMP269 | HDAC inhibitor |
| 85 | (R)-Ruxolitinib (INCB018424) | JAK inhibitor |
| 86 | Belinostat (PXD101) | HDAC inhibitor |
| 87 | Momelotinib (CYT387) | JAK inhibitor |
| 88 | IOX 2 | HIF inhibitor |
| 89 | Nexturastat A | HDAC inhibitor |
| 90 | Dacogen | DNA Methyltransferase inhibitor |
| 91 | JW55 | PARP inhibitor |
| 92 | RG108 | Histone Methyltransferase inhibitor |
| 93 | Chidamide | HDAC inhibitor |
| 94 | CAY10603 | HDAC inhibitor |
| 95 | RGFP 966 | HDAC inhibitor |
| 96 | MS436 | BET bromodomain |
| 97 | RVX-208 | Epigenetic Reader Domain inhibitor |
| 98 | MC1568 | HDAC inhibitor |
| 99 | PHOB | HDAC inhibitor |
| 100 | Rocilinostat (ACY-1215) | HDAC inhibitor |
| 101 | GSK2801 | Epigenetic Reader Domain inhibitor |
| 102 | Lomeguatrib | DNA Methyltransferase inhibitor |
| 103 | Veliparib (ABT-888) | PARP inhibitor |
| 104 | Nedaplatin | DNA/RNA Synthesis inhibitor |
| 105 | SRT1720 | Sirtuin inhibitor |
| 106 | Mocetinostat (MGCD0103) | HDAC inhibitor |
| 107 | Roxadustat (FG-4592) | HIF inhibitor |
| 108 | BG45 | HDAC inhibitor |
| 109 | C7280948 | Histone Methyltransferase inhibitor |
| 110 | AG-490 (Tyrphostin B42) | EGFR inhibitor; JAK inhibitor; HER2 inhibitor |
| 111 | AZD1208 | Pim inhibitor |
| 112 | CAY10683 (SantacruzaMate A) | HDAC inhibitor |
| 113 | OF-1 | BET bromodomain inhibitor |
| 114 | I-BET151 (GSK1210151A) | BET bromodomain inhibitor |
| 115 | CPI-203 | BET bromodomain inhibitor |
| 116 | AZD2461 | PARP inhibitor |
| 117 | C646 | Histone Acetyltransferase inhibitor |
| 118 | Baricitinib (LY3009104, INCB028050) | JAK inhibitor |
| 119 | LAQ824 (Dacinostat) | HDAC inhibitor |
| 120 | UNC0631 | Histone Methyltransferase inhibitor |
| 121 | KW2449 | FLT3 inhibitor |
| 122 | Tenovin-3 | Sirtuin inhibitor |
| 123 | CCT137690 | Aurora Kinase inhibitor |
| 124 | SNS-314 Mesylate | Aurora Kinase inhibitor |
| 125 | SGI-1776 free base | Pim inhibitor; FLT3 inhibitor |
| 126 | Gandotinib (LY2784544) | JAK inhibitor; FLT3 inhibitor; FGFR inhibitor |
| 127 | MI-2 (Menin-MLL Inhibitor) | Histone Methyltransferase inhibitor |
| 128 | MI-3 (Menin-MLL Inhibitor) | Histone Methyltransferase inhibitor |
| 129 | BAY85-3934;Molidustat | HIF inhibitor |
| 130 | XL019 | JAK inhibitor; PDGFR inhibitor; FLT3 inhibitor; c-Kit inhibitor |
| 131 | Barasertib (AZD1152-HQPA) | Aurora Kinase inhibitor |
| 132 | BIX 01294 Trihydrochloride | Histone Methyltransferase inhibitor |
| 133 | Tubastatin A BASE | HDAC inhibitor |
| 134 | Filgotinib (GLPG0634) | JAK inhibitor; Tyk inhibitor |
| 135 | EPZ005687 | Histone Methyltransferase inhibitor |
| 136 | GSK-525762A(I-BET-762) | Epigenetic Reader Domain inhibitor |
| 137 | BRD4770 | Histone Methyltransferase inhibitor |
| 138 | WP1066 | JAK inhibitor; STAT inhibitor |
| 139 | JIB-04 (NSC 693627)(E) | Histone Methyltransferase inhibitor |
| 140 | Inauhzin | Sirtuin inhibitor |
| 141 | CI994 (Tacedinaline) | HDAC inhibitor |
| 142 | SGI-1027 | DNA Methyltransferase inhibitor |
| 143 | Pracinostat (SB939) | HDAC inhibitor |
| 144 | GSK1324726A (I-BET726) | Epigenetic Reader Domain inhibitor |
| 145 | GSK343 | Histone Methyltransferase inhibitor |
| 146 | PFI-1 (PF-6405761) | BRD4 inhibitor |
| 147 | SGC-CBP30 | CREBBP/EP300 inhibitor |
| 148 | EPZ015666 | Histone Methyltransferase inhibitor |
| 149 | Remodelin hydrobromide | Histone Acetyltransferase inhibitor |
| 150 | Sirtinol | Sirtuin inhibitor |
| 151 | Histone Deacetylase Inhibitor IV | histone deacetylase inhibitor |
| 152 | WDR5-0103 | Histone methyltransferase |
| 153 | Dimethyloxaloylglycine (DMOG) | HIF inhibitor |
| 154 | Tofacitinib (CP-690550,Tasocitinib) | JAK inhibitor |
| 155 | Delapril Hydrochloride | ACE inhibitor |
| 156 | AG-14361 | PARP inhibitor |
| 157 | Sotrastaurin | PKC inhibitor |
| 158 | NVP-TNKS656 | PARP Inhibitor |
| 159 | NU7441 (KU-57788) | DNA-PK inhibitor |
| 160 | RG2833 (RGFP109) | HDAC inhibitor |
| 161 | UNC0638 | Histone methyltransferase(HMTase) Inhibitor |
| 162 | ITSA1 | TSA inhibitor |
| 163 | Chelerythrine chloride | PKC inhibitor |
| 164 | Trichostatin A (TSA) | HDAC inhibitor |
| 165 | AZ 960 | JAK inhibitor |
| 166 | GSK2879552 | Histone Demethylase |
| 167 | INO-1001 | PARP inhibitor |
| 168 | Mirin | ATM/ATR inhibitor; |
| 169 | A-366 | Histone Methyltransferase inhibitor |
| 170 | SGC707 | Histone Methyltransferase inhibitor; |
| 171 | Clevudine | DNA/RNA Synthesis inhibitor |
| 172 | EX 527 (Selisistat) | Sirtuin inhibitor |
| 173 | MLN8054 | Aurora Kinase inhibitor |
| 174 | Tubastatin A HCl | HDAC inhibitor |
| 175 | GSK6853 | Epigenetic Reader Domain |
| 176 | UNC 926 hydrochloride | L3MBTL1 inhibitor |
